# Taxonomic revision of grass frogs (Ptychadenidae, *Ptychadena*) endemic to the Ethiopian highlands

**DOI:** 10.3897/zookeys.1016.59699

**Published:** 2021-02-11

**Authors:** Sandra Goutte, Jacobo Reyes-Velasco*, Xenia Freilich, Abeje Kassie, Stephane Boissinot

**Affiliations:** 1 New York University Abu Dhabi, Saadiyat Island, Abu Dhabi, UAE New York University Abu Dhabi Abu Dhabi United Arab Emirates; 2 Department of Biology, Queens College, City University of New York, Flushing, New York, USA City University of New York New York United States of America; 3 Ethiopian Biodiversity Institute, Addis Ababa, Ethiopia Ethiopian Biodiversity Institute Addis Ababa Ethiopia; 4 Addis Ababa University, Addis Ababa, Ethiopia Addis Ababa University Addis Ababa Ethiopia

**Keywords:** Bioacoustics, herpetology, integrative taxonomy, linear morphometrics, phylogeny, species complex

## Abstract

Frogs of the genus *Ptychadena* that inhabit the Ethiopian highlands serve as a model system to understand biogeography, diversification, and adaptations to high elevations. Despite recent studies focusing on the systematics of this group, the taxonomy of the *Ptychadena neumanni* species complex remains only partially resolved, owing largely to the morphological resemblance of its members. Here, the taxonomy of this historically problematic group of frogs is revised by integrating morphological and molecular analyses on both century-old type specimens and more recently collected material. Based on these multiple lines of evidence, the *P. neumanni* species complex is shown to be more speciose than previously thought and four new species are described. With the aim of clarifying and stabilizing the taxonomy of the group, six species are also re-described and morphological and acoustic identification keys are provided. This study also establishes species distribution maps and reveals important differences in range size between the members of the *P. neumanni* complex, calling for adapted conservation measures across the Ethiopian highlands.

## Introduction

The grass frog genus *Ptychadena* Boulenger, 1917 currently contains 56 recognized species found throughout sub-Saharan Africa (Frost 2020). Some members of this group have dispersed to islands of the Indian (Madagascar, the Seychelles Islands and the Mascarene Islands) and Atlantic Oceans (Bioko island, Sao Tome; Vences et al. 2004; Measey et al. 2007), while others have invaded the Ethiopian highlands and can occur at elevations above 3000 meters. The genus *Ptychadena* is notorious for its highly conserved morphological features and the difficulty to distinguish closely related species and has thus baffled taxonomists for decades (e.g., Poynton 1970; Bwong et al. 2009; Dehling and Sinsch 2013). In particular, resolving the taxonomy of *Ptychadena* inhabiting the Ethiopian highlands has proven to be extremely challenging for a number of reasons, including the morphological resemblance among species, their inter- and intra-populations morphological variation, and confusing original descriptions (Ahl 1924). Thus, species misidentifications and taxonomic errors have accumulated throughout the literature (Perret 1980, 1994; Largen 2001; Smith et al. 2017a, b; Reyes-Velasco et al. 2018) and engendered confusion on the species’ ecology and distribution.

Six species of *Ptychadena* from the Ethiopian highlands were originally described based only on morphology: *P. neumanni* (Ahl, 1924), *P. erlangeri* (Ahl, 1924), *P. cooperi* (Parker, 1930), *P. nana* Perret, 1980, *P. harenna* Largen, 1997, and *P. wadei* Largen, 2000. A seventh species, *P. largeni*, was described by Perret (1994), but later synonymized with *P. neumanni* by Largen (2001). These species, except for *P. wadei*, form a monophyletic group, hereafter referred to as the *P. neumanni* complex (Freilich et al. 2014).

**Figure 1.**
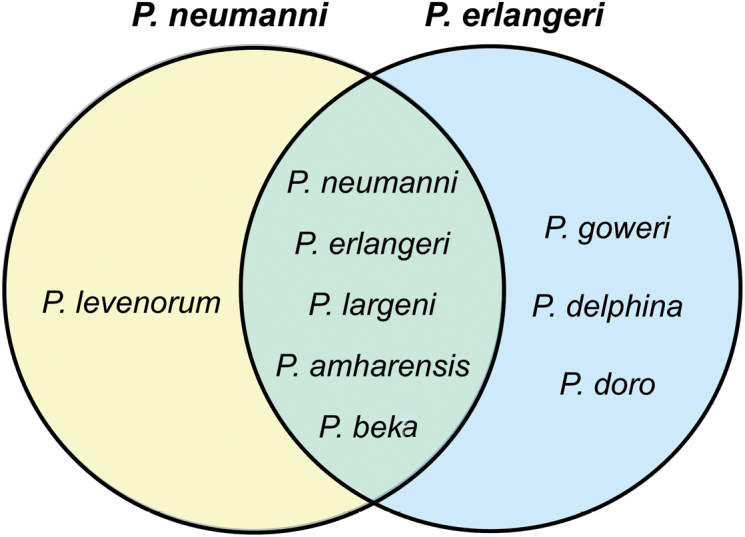
Species included under the names *Ptychadena neumanni* and *P. erlangeri* in previous studies. Species names within the circles correspond to the revised taxonomy. Left (yellow) and right (blue) circles contains all species identified as *P. neumanni* and *P. erlangeri*, respectively, since their original descriptions Ahl (1924). Species at the intersection of the two circles were identified as both species either in different studies (e.g., Largen 2001; Freilich et al. 2014; Smith et al. 2017a, b; Reyes-Velasco et al. 2018) or within the same study (e.g., Largen 2001). The full taxonomic history of the group is summarized in Suppl. material 3: Table S6.

Most of the confusion regarding the taxonomy of the Ethiopian *Ptychadena* arose from the original descriptions of *P. neumanni* and *P. erlangeri*, described by Ahl (1924) in the same article. While the description of *P. erlangeri* was based on a single female, the description of *P. neumanni* was based on 35 syntypes originating from four distinct localities. Perret (1980) showed that the type series of *P. neumanni* in fact contained multiple species, restricted *P. neumanni* to three males within the original type series and designating them as syntypes. Due to the sexual dimorphism present in the group, comparing the two species based on the morphological characters of their type specimens (one female versus three males) became very challenging, and numerous species misidentification occurred in the following studies (e.g., Perret 1994; Largen 2001; Smith et al. 2017; Reyes-Velasco et al. 2018). For example, the distribution maps of *P. erlangeri* and *P. neumanni* published by Largen (2001) and later Largen and Spawls (2010) each contained individuals of both species, as well as additional ones (Fig. 1, Suppl. material 3: Table S6). Because of the difficulty to identify the different species, some subsequent authors relied on these maps to assign names to the populations they encountered (e.g., Smith et al. 2017) and taxonomic confusion grew further.

Molecular analyses using both mitochondrial and nuclear loci revealed that *Ptychadena neumannisensu*Largen (2001) in fact comprised five distinct taxa, which did not form a monophyletic group (Freilich et al. 2014). Freilich and colleagues (2014) did not describe the potential new species they identified because they were not able to compare their specimens with the type specimens of previously described taxa. A subsequent publication reproduced Freilich and colleagues’ molecular analysis with a few additional samples and assigned names to these new taxa, but without any comparison with type specimens of previously described species and little morphological analysis (Smith et al. 2017a, b). Smith and colleagues thus assigned names of previously described species based solely on the locality of the few specimens they collected (Smith et al. 2017a, b), thereby adding to the general confusion regarding the taxonomy of this group (Suppl. material 3: Table S6).

**Figure 2.**
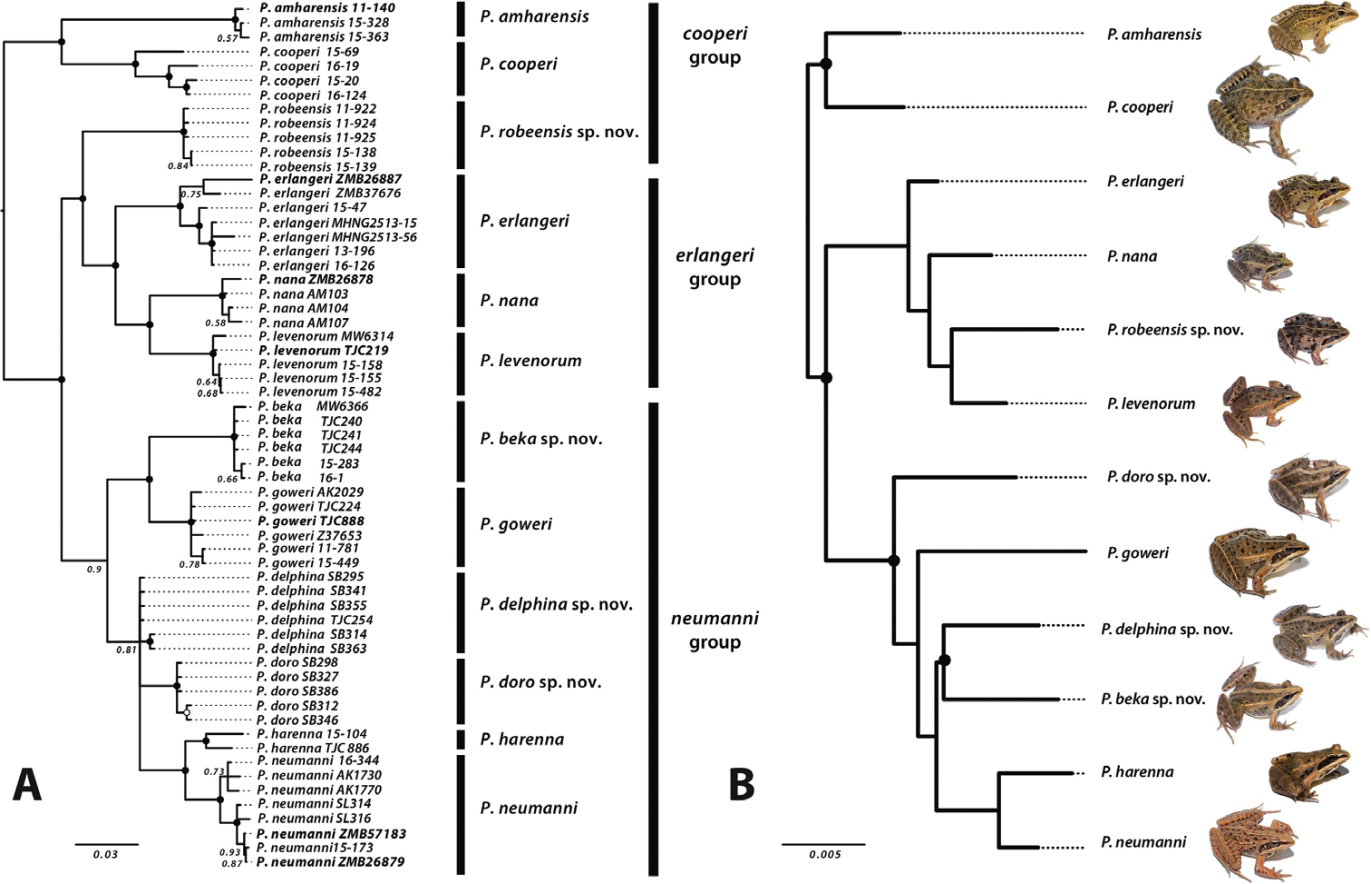
Phylogeny of the *Ptychadena neumanni* species complex **A** bayesian phylogenetic inference based on the concatenated sequences of the mitochondrial loci 12S and 16S rRNA as well as the protein-coding gene COX1. Black circles represent nodes with a posterior support of 1. Type specimens are indicated in bold **B** maximum Likelihood estimate (ML) of phylogenetic relationships in the *Ptychadena neumanni* species complex, inferred from a concatenated SNP dataset obtained using ddRAD sequencing. Black circles represent nodes with > 95% bootstrap support. Inset images represent members of each species: frogs are illustrated to the same scale. Modified from Reyes-Velasco et al. 2018.

In order to resolve the taxonomy of the *Ptychadena neumanni* species complex, we examined the type series of *P. neumanni*, *P. largeni*, *P. erlangeri*, and *P. nana*, and compared them morphologically to recently collected specimens for which molecular data was available (Reyes-Velasco et al. in review). We also sequenced mitochondrial DNA of the type specimens of these four species and included them in a phylogenetic analysis, which included recently sampled specimens as well as the type specimens of the species described by Smith and colleagues (2017a, b) (Fig. 2; Reyes-Velasco et al. in review). We recovered all the clades found in previous molecular phylogenetic analyses (Freilich et al. 2014; Smith et al. 2017a, b; Reyes-Velasco et al. 2018) and were able to determine the phylogenetic relationships of the historical type specimens relative to recently collected material, which allowed us to assign taxonomically correct names to these clades.

Our analyses grouped together the holotypes of *P. largeni* and *P. erlangeri*, showing that *P. largeni* was a junior synonym of *P. erlangeri* (Fig. 2). Additionally, these specimens grouped with the population designated as *P.* cf. *neumanni 2* by Freilich et al. (2014). The type specimen of *P. neumanni* grouped with the population considered as *P. erlangeri* by multiple authors since Largen (1997) (Suppl. material 3: Table S6). The population assigned to *P. neumanni* by Smith et al. (2017a, b) (*P.* cf. *neumanni 1* in Freilich et al. 2014), however, represented a new species. Finally, we showed that the type specimens of *P. nana* grouped with the population found in the Didda plateau, and that the individuals found on the east Arussi plateau (east of the Bale Mountains) represented a new species (Fig. 2; Reyes-Velasco et al. in review).

Based on molecular and morphological analyses, we describe four new species and re-describe six species of the *P. neumanni* complex with the goal of clarifying and stabilizing the taxonomy of the group. We also describe the advertisement calls of eleven species of the complex, and provide morphological and acoustic identification keys (Suppl. material 1: Appendix S1 and Suppl. material 2: Appendix S2). We did not include *Ptychadena wadei* in the current study, as this species is more distantly related to and readily distinguishable from other *Ptychadena* species from the Ethiopian highlands (Mengistu 2012). We also excluded *P. cooperi* and *P. harenna* from this revision, as their original descriptions are sufficiently complete to distinguish them from other species of the complex, however, we have included these three species in our identification key.

## Materials and methods

### Sampling

**Figure 3.**
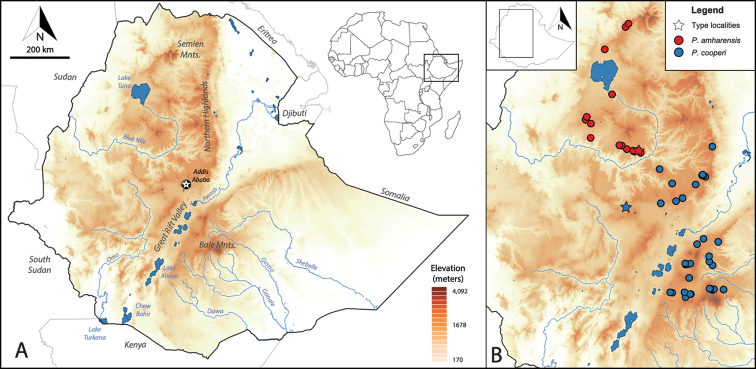
Distribution map of members of the *cooperi* species group in the highlands of Ethiopia **A** map of Ethiopia showing some of the most important geographic features of the country **B** distribution maps of members of the *cooperi* species group: *Ptychadena cooperi* (blue circles) and *P. amharensis* (red circles). Type localities for each species are indicated with a star.

**Figure 4.**
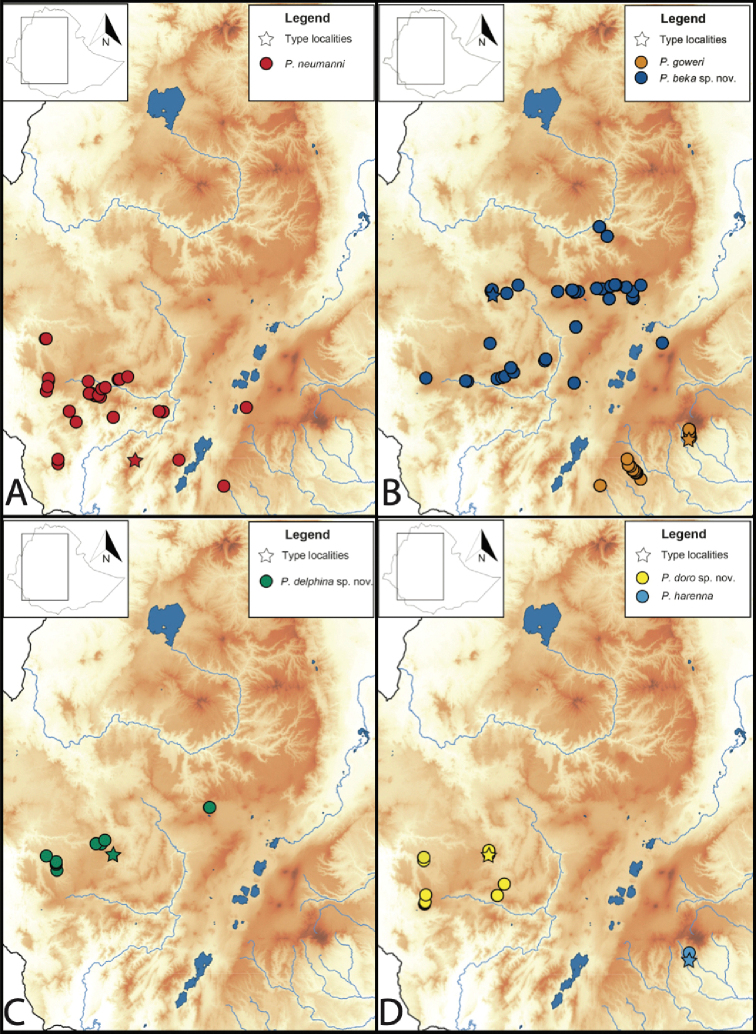
Distribution maps of members of the *neumanni* species group **A***Ptychadena neumannisensu stricto* (red circles) **B***Ptychadena beka* sp. nov. (blue circles) and *P. goweri* (orange circles) **C***Ptychadena delphina* sp. nov. (green circles) **D***Ptychadena doro* sp. nov. (yellow circles) and *P. harenna* (light blue circles). Type localities for each species are indicated with a star.

**Figure 5.**
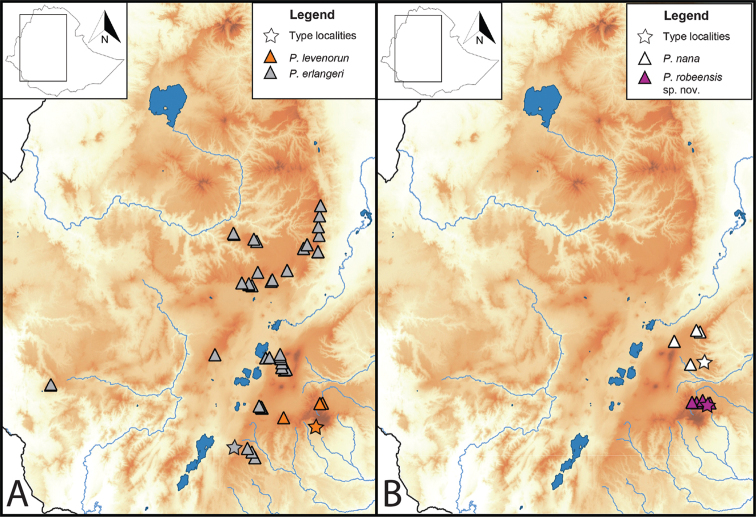
Distribution maps of members of the *erlangeri* species group **A***Ptychadena erlangerisensu stricto* (gray triangles) and *P. levenorum* (orange triangles) **B***Ptychadena robeensis* sp. nov. (purple triangles) and *P. nana* (white triangles). Type localities for each species are indicated with a star.

Methods of sampling are discussed in detail in Freilich et al. (2014) and Reyes-Velasco et al. (2018). In brief, we collected individuals of the *Ptychadena neumanni* species complex from the highlands of Ethiopia between 2011 and 2019 (Fig. 3, Suppl. material 3: Table S1). Our study was approved by the relevant Institutional Animal Care and Use Committee at Queens College and New York University School of Medicine (IACUC; Animal Welfare Assurance Number A32721–01 and laboratory animal protocol 19–0003). Frogs were sampled according to permits DA31/305/05, DA5/442/13, DA31/454/07, DA31/192/2010, DA31/230/2010, DA31/7/2011 and DA31/02/11 provided by the Ethiopian Wildlife Conservation Authority. We photographed individuals in life and euthanized them by ventral application of 20% benzocaine gel. We extracted tissue samples and stored them in RNAlater or 95% ethanol. Adult individuals were fixed in 10% formalin for 24 to 48 hours, and then transferred to 70% ethanol. After preservation, we took additional photographs of all individuals. All specimens were deposited at the Zoological Natural History Museum (**ZNHM**), Addis Ababa, Ethiopia. Tissue samples are deposited at the Vertebrate Tissue Collection, New York University Abu Dhabi (**NYUAD**). Species distribution maps for the 12 species of the complex are shown in Figs 3–5.

### Phylogenetic analyses

We describe the extraction of genomic DNA from fresh samples in Freilich et al. (2014) and Reyes-Velasco et al. (2018) and of mitochondrial genome of the type specimens of *Ptychadena neumanni*, *P. erlangeri*, *P. largeni*, and *P. nana* in Reyes-Velasco et al. (in review). Genetic data was already available for the type specimens of the more recently described species in the group (*P. amharensis*, *P. goweri*, and *P. levenorum*), so we did not re-sequence those type specimens. Fig. 2 reproduces the phylogeny obtained by Reyes-Velasco et al. (in review) based on the 12s rRNA, 16s rRNA and Cytochrome C oxidase I (COX1) mitochondrial genes for the type specimens as well as for recently collected specimens. GenBank accession numbers are given in Suppl. material 3: Table S2.

### Morphometric measurements

**Figure 6.**
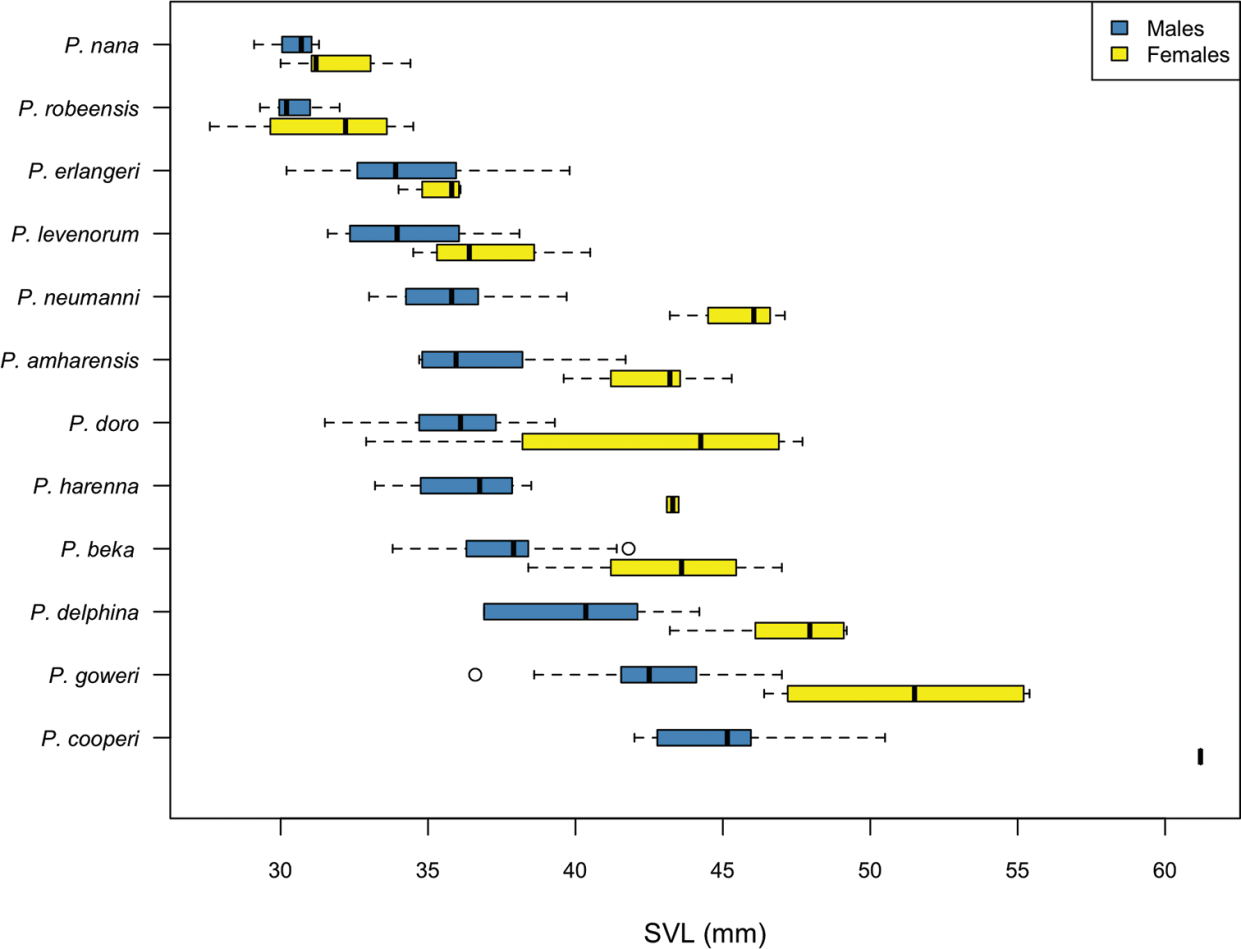
Snout-vent length (mm) of adult males and females of the *Ptychadena neumanni* complex.

We measured individuals that were collected in recent years as well as type specimens for all taxa in the *Ptychadena neumanni* species complex, including the species described by Smith et al (2017a, b), using a SPI dial caliper, model #31-415-3 (accuracy ± 0.0015 mm). We measured type specimens at the following institutions: Zoological Natural History Museum, Addis Ababa University, Ethiopia (**ZNHM**), The Natural History Museum (formerly British Museum, Natural History; **NHMUK**), Muséum d‘Histoire Naturelle, Geneva, Switzerland (**MHNG**) and the Museum für Naturkunde Berlin, Germany (**ZMB**). We took 19 linear morphometric measurements for each specimen (Fig. 6, Table 1, Suppl. material 3: Table S3), which are defined in Watters et al. (2016) and were shown to be useful for morphological differentiation of anurans.

List of abbreviations:

**ED** eye diameter;

**EN** eye-nostril distance;

**ETD** eye-tympanum distance;

**FinDW** longest finger disc width;

**FL** foot length;

**FLL** forearm length;

**GRV** great rift valley;

**HAL** hand length;

**HL** head length;

**HW** head width;

**IND** inter-nares distance;

**IOD** inter-orbital distance;

**MTL** metatarsal tubercle length;

**NS** snout-nostril distance;

**SL** snout length;

**SVL** snout-vent length;

**THL** thigh length;

**TD** tympanum diameter;

**TL** tibia length;

**Toe4DW** fourth toe disc width;

**UEW** upper eyelid width.

### Statistical analyses of linear morphometric measurements

We analyzed males and females separately due to sexual size dimorphism. We used the R package *FactoMineR* (Lê et al. 2008). Because of shrinkage due to variable conditions of fixation and long-term preservation of type specimens, we ran discriminant analyses on recently collected individuals only, in order to select the measurements best discriminating between species (removing the types of *Ptychadena neumanni*, *P. erlangeri*, *P. largeni*, and *P. nana*). We then compared type specimens to the results.

To determine the best discriminating morphometric measurements, we first split the 12 species into groups based on their body size using the snout-vent length (SVL): we ran an ANOVA followed by a Tukey Honest Significant Differences (Tukey HSD) test on log-transformed SVL measurements of the 12 species, of males and females separately. Species that were not significantly different in body size in both sexes were placed in the same group. We then ran discriminant analyses separately to select the best discriminating measurements in each of these groups, and an ANOVA followed by a Tukey HSD on these variables for each of these groups. Suppl. material 3: Tables S4, S5 show the results of statistical analyses on linear morphometric measurements.

Morphometric measurements were all log-transformed prior to analysis in order to approach normality. In order to correct for body size in our measurements, we used ratios of measurements / SVL. We did not use other adjustment method such as the one proposed by Lleonart et al. (2000) and used by others (e.g., Onn et al. 2018) to correct for allometric growth, because this method relies on coefficients calculated on populations and therefore artificially segregates individuals in a priori-determined groups. In addition, this method requires to measure multiple individuals of a given population before calculating the adjusted variables, which is not always possible in the field. Given that our species are found in sympatry and that our goal here is to define characters that may be used for species identification without any a priori, we chose to resort to a size correction only based on the individual’s own mensuration. Additionally, when comparing both correction methods on barcoded individuals, we found only marginal differences in the results and the ratios method proved to be more conservative.

### Recording and analysis of advertisements calls

Spontaneously calling males were located acoustically or visually at night between 18:00 and 05:00. The call type most often heard from single males was considered as the advertisement call. Other call types produced by conspecific males, often heard when males were close to each other or physically engaged were considered as aggressive or release calls (Bogert 1960) and were disregarded. We recorded advertisement calls in situ at a distance of 0.5–2 meters to avoid near-field effect (Rossing 2007) or excessive attenuation or distortion of the sound. We used a Sennheiser ME66 directional microphone with a Sennheiser K6 powering module and an Olympus LS-100 or a Marantz PMD661 MKII recorder at a sampling rate of 44.1 kHz at 16 bits. Comments were recorded at the end of each recording using a Sennheiser ME62 microphone. For each recording, the maximum Sound Pressure Level (SPL) of the call was measured with a Galaxy Audio CM-170 SPL meter on A-weighting (precision: 1 dB at 1 kHz). The exact distance between the microphone and the calling male was measured with a Leica E7100i laser meter (precision: 3 mm) subsequent to the capture of the frog.

When possible, video recordings were taken simultaneously with an infrared camcorder (SONY DCR-SR85) and custom-made Colorado Para Tech infrared lights to ensure the identity of the focal individual. Videos were subsequently used to select the focal male’s calls in recordings containing vocalizations of multiple individuals.

Advertisement calls were analyzed using Avisoft SAS (Sprecht 2017). We use a note-centered terminology scheme as described in Köhler et al. (2017), where the call constitutes a coherent unit and may contain one or several sub-units (notes), which, in turn, may contain distinct or indistinct pulses. We extracted 12 temporal and four spectral acoustic traits from our audio recordings: call duration, number of notes per call, note duration, inter-note interval duration (when applicable), note repetition rate (when applicable), number of pulses per note (when applicable), pulse duration (when applicable), inter-pulse interval duration (when applicable), pulse rate (when applicable), number of pulse groups per note (when applicable), number of pulses per pulse group (when applicable), relative time of peak amplitude, call peak frequency, call frequency bandwidth, minimal and maximal call frequencies. We did not extract values for call repetition rate or inter-call interval duration as these variables were highly dependent on the number of acoustically active individuals, and as such, are not taxonomically relevant characters.

**Figure 7.**
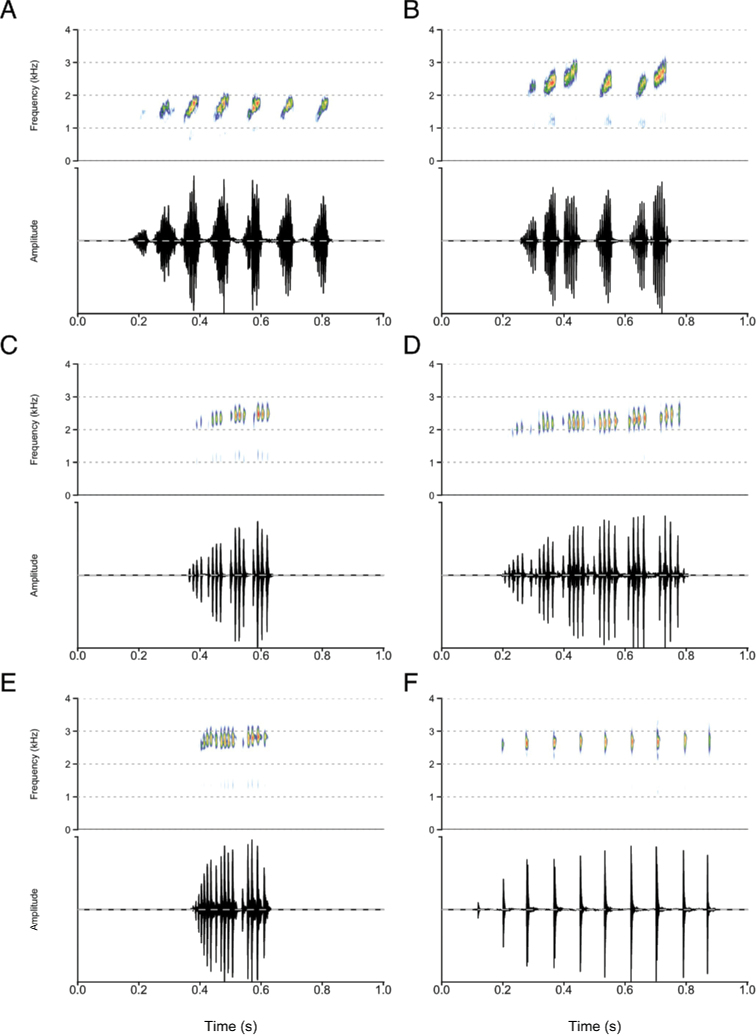
Spectrograms and oscillograms of calls of the *cooperi* and *erlangeri* species groups **A***P. cooperi***B***P. amharensis***C***P. erlangeri***D***P. levenorum***E***P. nana***F***P. robeensis* sp. nov.

**Figure 8.**
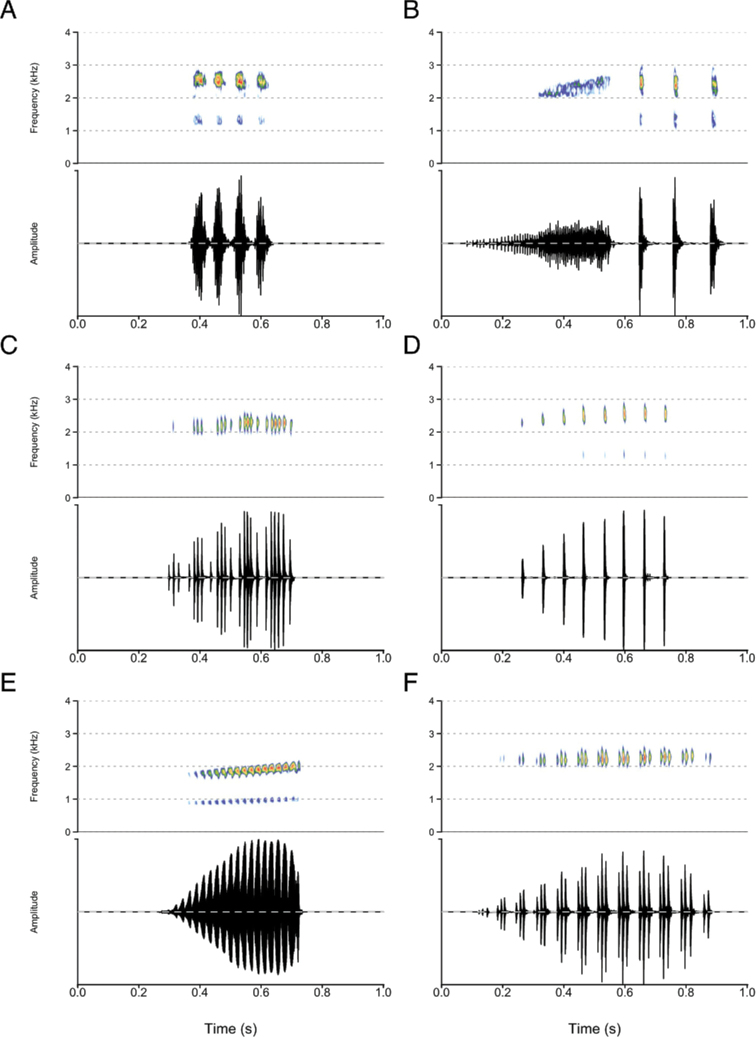
Spectrograms and oscillograms of calls of the *neumanni* species group **A***P. neumanni* call type **A, B***P. neumanni* call type **B, C***P. beka* sp. nov. **D***P. delphina* sp. nov. **E***P. doro* sp. nov. **F***P. goweri*.

Notes, pulses, and pulse groups were labelled semi-automatically using the pulse train analysis function and subsequently adjusting labels by eye. Sampling frequency was adjusted to 22050 Hz. Spectral traits were extracted from the spectrogram using the automatic parameter measurement function on spectrograms using a Fast Fourier Transformation (FFT) length of 512, Hamming windowing, 50% frame size, and 99.43% overlap between contiguous windows. All values were exported and averaged per individual and then per species in the R environment (R Core Team 2020). Spectrograms and oscillograms of the calls were plotted using the R package *seewave* (Sueur et al. 2008; Figs 7, 8).

## Results

### Phylogenetic analysis

The sampled populations of highland Ethiopian *Ptychadena* represent 12 distinct species grouped in three species groups, in agreement with previous studies (Freilich, Tollis, and Boissinot 2014; Reyes-Velasco et al. 2018; Reyes-Velasco et al. in review). The *cooperi* species group comprises *P. cooperi* and *P. amharensis*. Although the two species are morphologically distinct, they share call traits which distinguish them from the other species of the *P. neumanni* complex: their calls are composed of a few, rapidly repeated pulsed note, which presents an ascending frequency modulation and indistinct pulses (Figs 7, 8). The *neumanni* species group comprises six species: *P. neumanni*, *P. harenna*, *P. goweri*, *P. beka* sp. nov., *P. delphina* sp. nov., and *P. doro* sp. nov. Members of this group are generally larger and have longer hindlimbs and larger tympanums than members of the *erlangeri* species group. Sexual dimorphism is also more marked in this group (Fig. 6). Advertisement calls of the members of this group are very diverse and encompass all call types found in the *P. neumanni* species complex. The *erlangeri* species group comprises four species: *P. nana*, *P. erlangeri*, *P. levenorum*, and *P. robeensis* sp. nov. The members of the *erlangeri* species group are generally smaller and have shorter hind limbs, head, and snout than the members of the *cooperi* and the *neumanni* species groups (Fig. 6). They generally have smaller eyes and a shorter inter-nares distance and a smaller tympanum than the members of the other two species groups (Table 1). The sexual dimorphism is not as marked in this group as in the *neumanni* and *cooperi* species groups, with females’ body size range largely overlapping males’ body size range (Fig. 6). Members of this group present an important level of color polymorphism compared to the other species groups, with individuals of the same species presenting either brown, bright green, yellow or dark red background coloration or patterns. The vertebral stripe is also polymorphic in all species of this group, and can be absent, thin, medium, or wide. Advertisement calls of the members of the *erlangeri* species group are single notes composed of distinct pulses that may or may not be grouped within the note. Within *P. erlangeri*, two subclades emerge in molecular phylogenetic analyses, corresponding to the populations west and east of the GRV, respectively (Freilich et al. 2016).

### Linear morphometrics

Based on body size, we split the 12 species into three groups: small (*P. nana*, *P. robeensis* sp. nov., *P. levenorum* and *P. erlangeri*), large (*P. cooperi*, *P. goweri* and *P. delphina* sp. nov.), and medium to large (*P. amharensis*, *P. levenorum*, *P. erlangeri*, *P. beka* sp. nov., *P. doro* sp. nov., *P. neumanni*, *P. harenna*, and *P. goweri*). Certain species were placed in two groups because of the important body size variation within their range. Overall, males generally showed more significant differences than females, which may in some cases be due to low sample size for females. Results of the analyses are provided Suppl. material 3: Tables S4, S5. An identification key is provided as Suppl. material 1: Appendix S1.

#### Small size group: *P. erlangeri*, *P. levenorum*, *P. nana*, and *P. robeensis* sp. nov.

Within the *small* group, male *P. erlangeri* differed from all three other species in having significantly longer hind limbs (TL, THL, and FL/TL; Suppl. material 3: Table S3). They further differed from *P. nana* and *P. robeensis* males in SVL, FL, HAL, TD, and ED. Male and female *P. levenorum* differed from *P. nana* and *P. robeensis* sp. nov. in a larger body size and longer tibias. Male *P. levenorum* differed from *P. erlangeri* in head shape (SL, EN, IOD) and hind limbs (TL, THL, FL/TL, FL/THL). Female *P. levenorum* also had longer metatarsal tubercles than all other three species (Table 1).

**Table 1.** Morphometric measurements (average ± standard deviation) of the *Ptychadena neumanni* complex.

Species	Sex	N	SVL	HW	HL	SL	NS	IND	EN	IOD	ETD	TD	ED	UEW	FLL	HAL	FinDW	THL	TL	FL	Toe4DW	MTL
*P. amharensis*	M	6	36.9±2.7	13.9±1.4	13.2±0.6	5.5±0.3	2.8±0.2	2.9±0.3	2.5±0.3	2.1±0.2	1.2±0.2	2.9±0.3	4.2±0.6	2.7±0.4	6.8±0.3	8.3±0.5	0.6±0.1	17.4±1.2	18.8±1.3	18.9±0.9	0.6±0.1	1.6±0.3
	F	7	42.5±2	13.9±0.9	14.2±0.9	6.1±0.3	3±0.5	3.3±0.2	2.6±0.3	2.4±0.3	1.5±0.3	2.9±0.3	4.3±0.4	2.9±0.2	7.6±0.6	8.5±0.5	0.6±0.1	19.2±1.3	21.2±1.3	21.1±1.3	0.5±0.2	1.7±0.2
*P. beka*	M	9	37.9±2.6	14.5±1	14.2±1.3	5.7±0.4	2.9±0.4	3±0.2	2.6±0.3	2.7±0.3	1.1±0.4	2.9±0.2	4±0.5	2.6±0.4	6.7±0.5	8.3±0.5	0.6±0.1	17.6±1.2	19.5±1	19.5±1	0.6±0.1	1.7±0.2
	F	8	43.2±2.9	14.7±1.2	15.6±1.3	6.7±0.5	3.4±0.4	3.7±0.3	3.2±0.3	3.2±0.5	1.5±0.2	3.2±0.3	4.5±0.7	2.9±0.3	8±0.7	9.5±0.5	0.6±0.1	20.9±1.8	24.7±1.9	23.6±1.4	0.7±0.2	1.9±0.1
*P. cooperi*	M	8	45±2.7	17.7±1.1	16.5±1.5	7.3±0.5	4.2±0.5	3.3±0.4	2.9±0.4	2.9±0.5	1.7±0.3	3.6±0.4	4.9±0.5	3±0.3	9.7±1.1	11.5±1.2	0.7±0.2	22.5±2.7	26.4±1.5	25.3±1.5	0.7±0.1	1.9±0.2
	F	1	61.2	19.9	20.7	7.6	3.8	4	3.1	3.4	2.2	4.6	5.8	4.8	11.4	12.6	0.9	29.4	31.9	31.1	1	2.5
*P. delphina*	M	6	40.1±2.9	14.7±0.4	14.3±0.8	6.2±0.3	3±0.2	3.2±0.4	2.8±0.6	2.5±0.3	1.3±0.2	3±0.4	4.2±0.6	2.9±0.2	8.1±0.5	8.9±0.9	0.6±0.1	18.8±0.8	21.4±1.5	21.6±2.6	0.5±0	1.5±0.2
	F	6	47.2±2.3	15.9±1.4	16.3±0.8	6.9±0.4	3.3±0.3	3.4±0.2	3.2±0.2	3.2±0.3	1.6±0.2	3.4±0.3	5.2±0.4	3.3±0.3	9.3±0.7	10.1±0.6	0.6±0.1	22.9±1.9	25.9±2.2	25.7±1.6	0.7±0.1	1.8±0.1
*P. doro*	M	13	35.8±2.2	12.6±0.8	13.1±1.1	5.6±0.4	2.7±0.3	3.1±0.2	2.5±0.2	2.8±0.4	1.1±0.2	2.5±0.3	4±0.4	2.5±0.2	7.1±0.5	8.5±0.7	0.6±0.2	17.5±1.6	20.7±1.4	21.1±2.2	0.5±0.1	1.5±0.3
	F	6	42.4±5.7	14.2±0.9	15±0.4	6.7±0.5	3.4±0.5	3.5±0.4	2.8±0.5	3.2±0.5	1.3±0.3	2.9±0.4	4.6±0.4	3±0.4	8.9±0.9	10.4±1	0.6±0.1	22.6±2.4	26.6±1.1	26.9±1.6	0.8±0.2	1.8±0.3
*P. erlangeri*	M	35	34.1±2.5	11.7±1	12.2±1.3	5±0.5	2.6±0.3	2.7±0.3	2.2±0.2	2.5±0.4	1±0.2	2.4±0.3	3.5±0.4	2.4±0.4	6.3±0.6	7.2±0.7	0.6±0.1	15.8±1.3	17.9±2	18.3±1.8	0.5±0.1	1.4±0.3
	F	10	34.6±1.7	11.6±1	12.2±1.3	5.2±0.5	2.7±0.3	2.9±0.2	2.5±0.2	2.5±0.4	1±0.2	2.3±0.3	3.6±0.5	2.4±0.3	6.2±0.5	7.5±0.8	0.5±0.1	16.9±2.1	18.7±2.5	19.2±1.8	0.5±0.1	1.4±0.3
*P. goweri*	M	15	42.4±2.7	15.7±0.9	16.2±1.4	6.7±0.5	3.1±0.3	3.5±0.4	3.1±0.4	3.2±0.4	1.5±0.2	3.1±0.4	4.3±0.7	2.9±0.2	8.5±0.9	10.2±0.8	0.7±0.2	21.9±1.5	25.4±1.5	25.7±1.2	0.7±0.1	2±0.3
	F	6	51.2±4.2	17.6±2	18.6±2.1	7.5±0.4	3.7±0.3	3.9±0.5	3.5±0.3	3.5±0.4	1.8±0.3	3.5±0.4	5±0.5	3.3±0.2	9.8±1	11.8±1	0.7±0.1	25.7±2.7	30.4±2.3	29.4±2	0.8±0.1	2.5±0.3
*P. harenna*	M	4	36.3±2.3	13.6±1.2	14.2±1.6	5.8±0.3	3.1±0.2	3.4±0.5	2.4±0.3	2.4±0.5	1.2±0.4	3.1±0.6	4.8±0.6	2.8±0.5	7.6±0.4	8.1±0.5	0.6±0.1	18.4±2	20.6±1.3	20.7±1.7	0.5±0.1	1.4±0.1
	F	2	43.3±0.3	15.4±1.2	16±0.8	7±0.4	3.4±0.3	3.8±0.1	3±0.1	4.2±0.2	1.4±0.1	3.1±0.4	4.7±0.1	2.7±0.6	7.8±0.5	9.8±0.6	0.8±0.4	21.8±0.4	24.9±0.4	24±0.5	0.8±0.3	2±0.5
*P. levenorum*	M	8	34.3±2.3	11.7±1.3	11.9±1.1	4.6±0.5	2.4±0.3	2.4±0.3	1.8±0.4	2.2±0.3	1±0.3	2.2±0.2	3.2±0.4	2.2±0.4	6.5±1	7±0.7	0.6±0.1	14.2±1.4	16.9±1.8	18±2	0.5±0.1	1.6±0.2
	F	4	37±2.5	12.2±0.9	12.1±0.5	4.8±0.5	2.5±0.3	2.7±0.4	2.1±0.2	2.6±0.3	1.3±0.2	2.4±0.4	3.5±0.6	2.5±0.4	6.6±0.8	7.5±0.5	0.5±0.2	16±1.6	18.9±2.1	20.1±2.4	0.6±0.1	1.8±0.2
*P. nana*	M	12	28.3±3.3	10.2±1.4	10.1±0.7	4.2±0.4	2.2±0.2	2.3±0.2	1.8±0.2	2.2±0.3	0.9±0.1	1.8±0.2	2.9±0.5	2±0.3	5±0.6	5.6±0.6	0.5±0.1	11.7±1.4	12.7±0.9	13.8±1	0.5±0.1	1.2±0.2
	F	12	29.6±3.2	9.5±1.3	10±0.8	4.2±0.4	2.3±0.3	2.5±0.3	1.9±0.2	2±0.4	1±0.2	1.8±0.2	3.2±0.4	2.1±0.4	4.9±0.3	5.8±0.6	0.5±0.1	12.2±1.2	12.9±1.1	14.3±1.2	0.5±0.1	1.2±0.2
*P. neumanni*	M	20	34.6±2	12±1.2	13.6±0.8	5.6±0.4	2.8±0.4	3.2±0.3	2.6±0.3	3.1±0.3	1±0.1	2.7±0.4	3.9±0.2	2.3±0.4	6.8±0.4	8.1±0.6	0.6±0.2	17.9±1.2	20.1±1.1	20.6±1.4	0.6±0.1	1.4±0.2
	F	8	45.6±1.4	15.2±1.1	16.7±1.6	7.1±0.4	3.6±0.1	4±0.4	3.2±0.3	3.6±0.4	1.5±0.3	3.4±0.4	4.7±0.5	3±0.4	8.3±0.7	9.8±0.7	0.7±0.2	21.8±1.5	26.1±0.8	26.4±1.4	0.7±0.2	1.9±0.3
*P. robeensis*	M	13	30.3±0.7	10.7±0.7	11.1±0.8	4.3±0.2	2.2±0.2	2.4±0.2	1.9±0.1	2.4±0.3	1.1±0.1	2±0.2	3±0.4	2.1±0.2	5.4±0.5	6.4±0.4	0.6±0.2	12.4±0.8	13.6±0.8	15.1±1	0.5±0.1	1.2±0.3
	F	4	31.6±2.9	11.1±0.6	10.9±0.2	4.3±0.4	2.2±0.5	2.5±0.2	2±0.1	2.5±0.4	1.1±0.2	2±0.2	3.3±0.1	2±0.3	5.5±0.5	6.5±0.5	0.5±0.1	12.2±1.1	13.9±0.8	15.4±1.2	0.6±0.1	1.3±0.2

**Table 2.** Call variables for pulsed calls. Durations are given in ms; frequencies, frequency bandwidths, and pulse rates are given in Hz (s^-1^).

Species	N individuals	N calls	Call duration	Number of notes	Pulse duration	Pulses per note	Overall Inter-pulse duration	Overall pulse rate	Groups per note	Group duration	Pulses per group	inter-pulse duration within groups	Pulse rate within group	Peak frequency	Min frequency	Max frequency	Fequency bandwidth	Relative time of peak amplitude
*P. beka*	5	128	410±113	1±0	3±0	24.5±5.7	14±4	62.4±14.1	10.5±2.6	21±17	3.7±1.9	9±1	210.8±129.7	2482±142	2169±118	2529±150	742±66	258±77
*P. delphina*	4	33	504±92	1±0	5±1	8.4±1.4	64±8	16.8±2.4	–	–	–	–	–	2327±147	1982±161	2366±166	736±94	384±129
*P. erlangeri*	5	226	290±35	1±0	4±0	16.5±1.6	15±1	57.1±4.1	4.3±0.8	37±17	4.1±2.5	11±2	123.8±92.7	2343±454	1709±383	2600±297	1138±767	230±14
*P. goweri*	4	32	634±74	1±0	3±0	33.5±2.9	17±3	53.6±8.4	14.1±2.3	20±14	3.3±1.5	9±2	231.6±182.1	2318±86	1934±57	2356±88	838±156	404±65
*P. levenorum*	2	30	465±98	1±0	3±0	23.5±4.3	17±3	51.1±5.6	7.1±1.5	36±25	4.1±1.5	12±2	172.7±142.6	2223±90	1840±51	2286±73	864±40	345±34
*P. nana*	5	56	205±47	1±0	4±0	16.9±2.8	9±2	84±10.2	2.3±0.8	76±49	7.2±4.7	9±2	123±71.3	2801±52	2461±26	2845±53	751±63	136±31
*P. robeensis*	5	101	534±53	1±0	4±0	9.8±1	57±5	18.5±1.8	–	–	–	–	–	2876±74	2397±40	2896±78	853±97	292±19

**Table 3.** Call variables for calls composed of several notes.

Species	N individuals	N calls	Call duration	Number of notes	Note duration	Inter-note duration	Note rate	Peak frequency	Min frequency	Max frequency	Frequency bandwidth	Relative time of peak amplitude
*P. cooperi*	3	54	393±126	4.5±1.2	42±8	58±11	11.7±0.8	1653±64	666±267	1687±62	1547±494	235±82
*P. amharensis*	4	47	355±276	5±3.5	43±15	37±12	14.7±3.6	2224±158	1535±274	2247±177	1282±176	169±44
*P. doro*	3	21	411±41	1±0	411±41	–	–	1966±105	1752±87	1999±93	415±31	295±39

**Table 4.** Call variables for *P. neumanni* two call types.

Species	N (individual)	Call type	N calls	Call duration	Note type	Number of notes	Note duration	Inter-note duration	Note rate	Peak frequency	Min frequency	Max frequency	Frequency bandwidth	Relative time of peak amplitude
*P. neumanni*	3	A	78	307±149	a	5.8±2.4	32±14	28±11	19.1±3.5	2406±339	2088±274	2408±332	703±119	19±8
	1	B	13	437±54	b	1±0	437±54	–	–	2207±129	1440±578	2369±178	1392±692	346±74
				244±67	c	3±0.6	19±5	94±5	12.6±1.3	2337±191	1975±263	2336±187	744±179	6±2

*Ptychadena nana* and *P. robeensis* sp. nov. were the most similar and challenging to distinguish based on morphometry alone. We therefore ran an additional analysis including only the two species. Males showed differences in head shape (HL, HW/HL, TD) and limb sizes (FL, HAL, FL/THL). Females showed differences in head shape (TD/ED, IND/IOD, IOD/ED) and forearm length (Suppl. material 3: Table S3).

#### Large size group: *P. cooperi*, *P. delphina* sp. nov., and *P. goweri*

Male *Ptychadena cooperi* differed in head shape (HW, NS, NS/SL) from *P. goweri* and *P. delphina* sp. nov. males (Table 1, Suppl. material 3: Table S3). They also differed in hand length (HAL), snout length (SL) and eye-tympanum distance (ETD) from *P. delphina* sp. nov. and head shape (HW/HL and TD) and forearm length (FLL) from *P. goweri*. *Ptychadena cooperi* was significantly larger than *P. delphina* sp. nov. in both males and females. Male *P. delphina* sp. nov. differed from *P. cooperi* and *P. goweri* in hind limb proportions (TL, MTL, Toe4DW). They further differed from *P. goweri* in hind limbs morphology (THL, TL, FL) and interorbital distance (IOD). Foot (FL) and metatarsal tubercle lengths (MTL) also differed between female *P. delphina* sp. nov. and *P. goweri* (Suppl. material 3: Table S3).

#### Medium size group: *P. amharensis*, *P. beka* sp. nov., *P. doro* sp. nov., *P. erlangeri*, *P. goweri*, *P. harenna*, *P. levenorum*, and *P. neumanni*

Male *Ptychadena levenorum* significantly differed from all other species in THL and EN (Suppl. material 3: Table S3). Additionally, they differed from all other species except *P. erlangeri* in snout shape (IND and SL). *Ptychadena goweri* males were larger than all other species except for *P. delphina* sp. nov. and had longer thighs than all species except for *P. delphina* sp. nov. and *P. harenna*. Males of *P. goweri* further differed from all other species, except *P. beka* sp. nov. and *P. doro* sp. nov., in their IOD. Finally, *P. goweri* differed from all species except *P. doro* sp. nov. and *P. harenna* in hind limb size (TL and FL), and all species but *P. beka* sp. nov. in their Toe4DW (Suppl. material 3: Table S3).

*Ptychadena neumanni* males differed from *P. amharensis*, *P. erlangeri*, *P. harenna*, *P. levenorum* and *P. delphina* sp. nov. in IOD. *Ptychadena neumanni* also differed from *P. goweri* in ETD, FLL, and MTL. *Ptychadena neumanni* also differed from *P. levenorum* and *P. erlangeri* in TD, from *P. goweri* and *P. levenorum* in HAL, and from *P. levenorum* in ED and THL (Suppl. material 3: Table S3). *Ptychadena erlangeri* males differed from *P. doro* sp. nov., *P. goweri*, *P. harenna* and *P. delphina* sp. nov. in FLL. They had a narrower head than *P. amharensis*, *P. beka* sp. nov., *P. goweri*, *P. delphina* sp. nov., and further differed in SL from *P. beka* sp. nov., *P. doro* sp. nov., *P. goweri*, *P. harenna* and *P. delphina* sp. nov. Finally, they differed from *P. doro* sp. nov. and *P. goweri* in HAL and FL. *Ptychadena beka* sp. nov. differed from *P. doro* sp. nov. in HW and hindlimbs length (TL and FL), from *P. amharensis* in IOD, from *P. delphina* sp. nov. in FLL (Suppl. material 3: Table S3).

Few significant differences were found between the morphometric measurements of males *P. harenna*, *P. delphina* sp. nov., *P. doro* sp. nov. and *P. amharensis* (Suppl. material 3: Table S5). We therefore ran an additional analysis on these four species alone. *Ptychadena amharensis* males differed from *P. doro* sp. nov., *P. harenna* and *P. delphina* sp. nov. in TL. They also differed from *P. harenna* and *P. delphina* sp. nov. in FLL, from *P. doro* sp. nov. in head shape (HW, IOD, IOD/ED, HW/HLW) and FL, and from *P. delphina* sp. nov. in SL. Female *P. amharensis* differed from *P. doro* sp. nov., *P. harenna* and *P. delphina* sp. nov. in IOD, from *P. doro* sp. nov. in limb length (TL, FL, HAL, FLL) and SL, from *P. delphina* sp. nov. in ED.

Head shape differed between males of *Ptychadena harenna* and of *P. doro* sp. nov. (TD, ED, IOD/ED; Suppl. material 3: Table S3) and females of *P. harenna* and of *P. delphina* sp. nov. (IOD and IOD/ED; Suppl. material 3: Table S3). Finally, *P. doro* sp. nov. males differed from *P. delphina* sp. nov. in SVL, HW, and TL (Suppl. material 3: Table S3).

### Acoustic analyses

Advertisement calls of the species of the *P. neumanni* complex were diverse (Figs 7, 8) and provided useful discriminant characters. Species of the group are thus more easily identified by their advertisement calls than their morphology. An identification key based on spectral and temporal call traits is provided as Suppl. material 2: Appendix S2. Three major types of advertisement calls are produced by the species of the *P. neumanni* complex: a group of a few short notes with indistinct pulses, a single note with partly fused pulses, and a single note comprising distinct pulses.

*Ptychadena cooperi*, *P. amharensis*, and *P. neumanni* produce calls composed of a few pulsed notes, somewhat resembling human laughter. The call of *P. neumanni* (call type A) differs from those the *P. cooperi* species group in that it does not contain any frequency modulation. The call of *P. cooperi* can be distinguished from the call of *P. amharensis* by its lower average dominant frequency, reflecting a larger body size.

*Ptychadena doro* sp. nov. produces an advertisement call very distinctive within the group, reminiscent of the sound produced by a chicken. It is a relatively long, single note with partly fused pulses and an increase of the dominant frequency along the note.

Most species of the *P. neumanni* complex (*P. erlangeri*, *P. levenorum*, *P. nana*, *P. robeensis* sp. nov., *P. beka* sp. nov., *P. delphina* sp. nov., and *P. goweri*) produce single-note calls containing distinct pulses. *Ptychadena robeensis* sp. nov. and *P. delphina* sp. nov. are distinct from the other species in that their pulses are evenly and well-spaced, while the other species calls contain multiple pulse groups. The size difference between the two species is reflected in the peak frequency of their calls, with *P. robeensis* sp. nov. calling at a higher frequency *than P. delphina* sp. nov. *Ptychadena goweri* produces the longest (634 ± 74 ms), while *P. nana* produces the shortest call (205 ± 47 ms) of the subgroup. The calls of *Ptychadena beka* sp. nov., *P. erlangeri* and *P. levenorum* can be distinguished based on the number of pulse groups and the number of pulses within each pulse group: the call of *P. beka* sp. nov. in composed of 8–13 pulses groups containing 2–6 pulses each, while *P. levenorum* produces calls with 5–9 pulse groups of 10–33 pulses each. *Ptychadena erlangeri* pulse structure is variable, with 3–6 pulses groups containing 3–21 pulses each, but can be distinguished from *P. levenorum* but a shorter call duration (< 325 ms versus > 370 ms for the call of *P. levenorum*).

Finally, *Ptychadena neumanni* produces a second call type (type B), containing a long, pulsed note followed by a short series of distinct pulses. This call type is unique within the *P. neumanni* species complex, and we believe that it may be an aggressive call. However, our data are insufficient to attribute the function of this second call type with certainty.

### Integrative taxonomy

Our analysis distinguishes 12 species in the *Ptychadena neumanni* complex, in agreement with previous molecular phylogenetic studies (Freilich et al. 2014; Reyes-Velasco et al. 2018). For eight of these species, names are available, while four are new species. Our previous molecular and morphometric analyses, including type specimens, demonstrated that the specimens designated as *P. erlangeri* by Largen 2001; Freilich et al. 2014; Smith et al. 2017a, b and Reyes-Velasco et al. 2018 belong to *P. neumanni* Ahl, 1924 (Reyes-Velasco et al. in review). Similarly, our results show that *P. largeni* described by Perret in 1994 is a junior synonym of *P. erlangeri* Ahl, 1924, and not of *P. neumanni* as stated by Largen (2001), nor a proper species as considered by Smith et al. 2017a, b (Reyes-Velasco et al. in review).

Smith et al. (2017a, b) assigned *P. neumanni* to the population designated as *P.* cf. *neumanni 1* in Freilich et al. (2014) without comparing the sequenced specimens to the type series of *P. neumanni*. Our results show that *P.* cf. *neumanni 1* constitutes in fact a new species, which we name *Ptychadena beka* sp. nov. and describe hereafter. The three new species described in Smith et al (2017a, b), *P. amharensis*, *P. goweri* and *P. levenorum*, based on molecular evidence constitute valid species names. However, no useful diagnostic character other than molecular data was provided in the original descriptions. We therefore re-describe those three species below.

## Systematic accounts

### Ptychadena amharensis

Taxon classificationAnimaliaAnuraPtychadenidae

Smith, Noonan & Colston, 2017

90E568FB-8FB5-5810-81B3-0227A31044BC

#### Type material.

***Holotype*.** by original designation. A juvenile (XF140) collected on 18 July 2011 by X. Freilich and S. Boissinot in Dejen, Amhara region, Ethiopia (10.1908°N, 38.140°E, 2425 m a.s.l.). ***Paratypes*.** Three adult females (XF141, XF142, XF143) collected by X. Freilich and S. Boissinot on the same date and location. All type specimens and material examined are deposited at ZNHM.

#### Material examined.

In addition to the type series, we examined one male (15–313) collected on 17 August 2015 by X. Freilich, J. Reyes-Velasco and S. Boissinot southeast of Debre Markos (10.2745°N, 37.8564°E, 2261 m a.s.l.), one female (15–367) collected on 19 August 2015 by X. Freilich J. Reyes-Velasco and S. Boissinot southwest of Debarq (13.0359°N, 37.799°E, 2583 m a.s.l.), one female (16–175) collected on 15 July 2016 by X. Freilich, J. Reyes-Velasco and S. Boissinot in Debre Markos (10.35195°N, 37.7414°E, 2803 m a.s.l.), one female (SB580) collected on 8 July 2018 by S. Goutte and Y. Bourgeois north-west of Debre Markos (10.3566°N, 37.6571°E, 2589 m a.s.l.), one female (SB584) collected on 8 July 2018 by S. Goutte and Y. Bourgeois in Debre Markos (10.35195°N, 37.7414°E, 2388 m a.s.l.) and five males (SB591, SB592, SB593, SB597 and SB606) collected on 9 July 2018 by S. Goutte and Y. Bourgeois in Debre Markos (10.3520°N, 37.7414°E, 2388 m a.s.l.).

#### Diagnosis.

**Figure 9.**
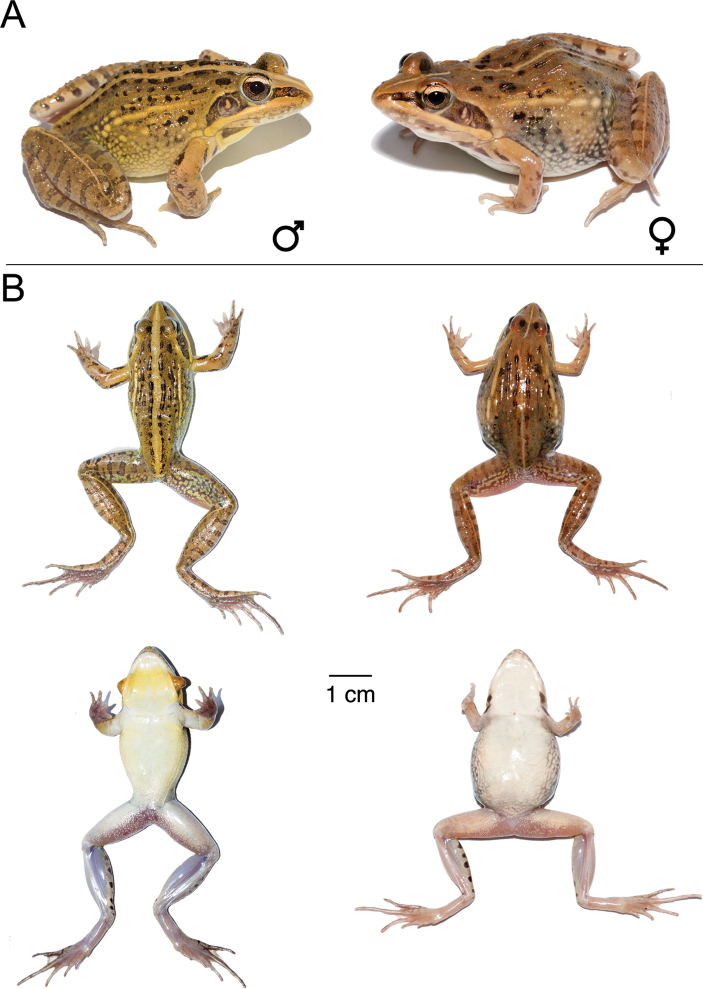
*Ptychadena amharensis***A** live male (SB593; left) and female (SB580, right) **B** dorsal and ventral views of the same individuals (male left, female right) after euthanasia and before fixation.

Medium-sized species (male (6) SVL 36.9 ± 2.7 mm, female (7) SVL 42.5 ± 2.0 mm) of the *cooperi* species group (Fig. 9). It differs from other members of the *Ptychadena neumanni* species complex by the following combination of characters: (1) tibia half of the snout-vent length, (2) eye close to one another (male IOD/HW 0.16 ± 0.02, female IOD/HW 0.17 ± 0.02), (3) vertical light stripe on the tympanum, (4) vocal sacs are light grey to cream, sometimes mottled with light grey, (5) adult males’ bodies covered in small warts.

#### Comparison.

*Ptychadena amharensis* is smaller than *P. cooperi* and larger than *P. nana* and *P. robeensis* sp. nov. It has shorter hindlimbs than *P. doro* sp. nov., *P. neumanni*, *P. goweri*, and *P. harenna*. *Ptychadena amharensis* has a shorter head than *P. beka* sp. nov., *P. neumanni*, *P. harenna* and *P. goweri*. The species’ interorbital distance is shorter than in *P. doro* sp. nov., *P. delphina* sp. nov., *P. erlangeri*, *P. levenorum*, *P. goweri*, *P. nana*, *P. neumanni*, and *P. robeensis* sp. nov.

#### Description of the holotype.

**Figure 10.**
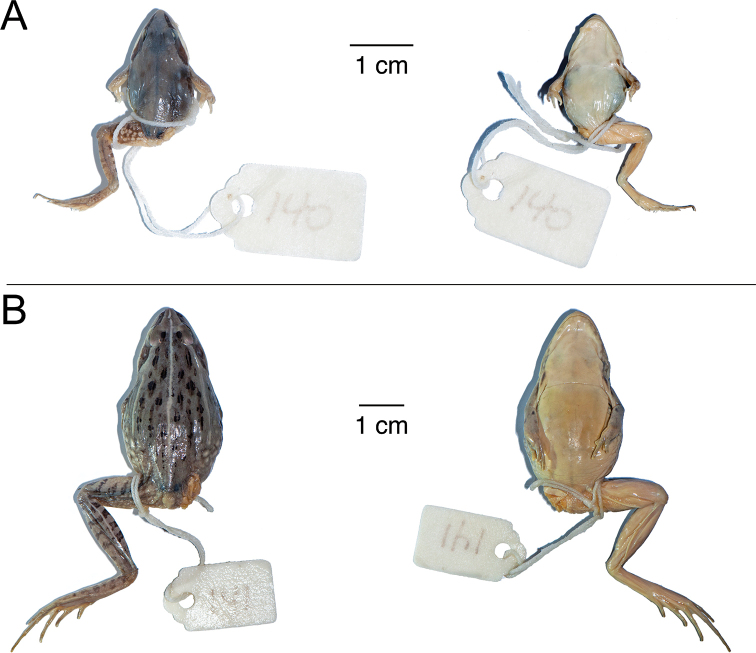
Type specimens of *Ptychadena amharensis***A** dorsal and ventral views of the juvenile holotype (XF140) **B** dorsal and ventral views of one of the three adult female paratypes (XF141).

Juvenile, in poor condition (Fig. 10). Right hind limb missing. No visible ridges or coloration on the back due to the poor condition of the specimen. Finger formula: 1<2<4<3, hand free of webbing. Toe formula: 1<2<5<3<4. Toe webbing indistinguishable. Tongue bifurcated and free for half is length. Maxillary and premaxillary teeth present, vomerine teeth not visible. Throat cream.

Measurements of the holotype (mm): SVL 21.4, HW 8.8, HL 9.3, SL 3.7, NS 2.1, IND 2.1, EN 1.8, IOD 1.6, ETD 0.9, TD 1.6, ED 2, UEW 1.1, FLL 3.9, HAL 4.9, F4DW 0.3, THL 9.6, TL 9.7, FL 10.1, T4DW 0.3, MTL 0.9.

#### Coloration of the holotype in preservative.

Dorsal background color is grey, with almost completely faded away dark grey markings in between the eyes and on the upper part of the dorsum. Dark brown canthal stripe from the tip of the snout to the back of the jaw. No vertical stripe or blotch on the tympanum. Upper lip, throat, and posterior part of flanks cream. Irregular dark brown markings on the anterior third of the flanks. Ventrum, ventral side of the thighs and tibias uniformly cream. Faint dark brown bars on the tibia. Thighs posteriorly dark brown with large cream spots.

#### Variations.

In life, background coloration varies from dark orange to light olive or yellowish brown. Small, more or less distinct, dark brown blotches distributed on the dorsal ridges and on the antero-dorsal part of the flanks. Cream or sand-colored thin or wide vertebral stripe present in all examined specimens.

Iris bicolored, with the upper third silver to sand color, and the lower two thirds brown or copper to dark brown. Upper and lower jaws with light brown irregular markings. Dark brown canthal stripe from the tip of the snout to the back of the upper jaw. More or less distinct cream-colored vertical stripe or blotch on the tympanum. Light thin stripe on the tibia, extending over the lower third of the thigh. Back thighs light yellowish grey to greenish yellow reticulated with dark grey. Ventrum white to light yellow, throat white to bright yellow. Vocal sacs cream or light grey sometimes molted with light grey. Dorsum and hindlimbs of adult males covered in small warts.

#### Habitat, distribution, and natural history.

*Ptychadena amharensis* is found in the Amhara plateau, at elevations ranging from 1824 m to 2642 m a.s.l. This species is found notably around Debre Markos, Enjebara, Bahar Dar and Gondar. The northern-most individuals were found just south of Debarq (13.1098°N, 37.8637°E), while the southernmost individuals were found around Dejen (10.1908°N, 38.1401°E), just North of the Blue Nile. This species has not been found south of the Blue Nile.

Males are found calling at night from flooded grass fields, sometimes aggregating in important numbers, in sympatry with *Ptychadena wadei* and *Ptychadena pumilio* south of Bahar Dar.

#### Advertisement call.

*Ptychadena amharensis* males produce calls (4 males, 47 calls) of 355 ± 276 ms duration, containing 4 ± 3.5 pulsed notes. Each note is 43 ± 15 ms in duration and pulses are indistinct. Call rate is highly dependent of the social context as *P. amharensis* tends to call in large choruses and their motivation to call is linked to the number of acoustically active conspecific males in their direct surroundings. Call dominant frequency is 2224 ± 158 Hz. Notes are frequency modulated, with an increase in dominant frequency within each note.

Within the *P. neumanni* complex, the call of *P. amharensis* can be distinguished from those of *P. erlangeri*, *P. levenorum*, *P. nana*, *P. robeensis* sp. nov., *P. beka* sp. nov., *P. delphina* sp. nov., *P. goweri*, and *P. neumanni* call type 2 by the indistinct pulses of its notes. It is distinct from *P. doro* sp. nov. by having calls composed of multiple notes, a higher dominant frequency and wider frequency bandwidth. The call of *P. amharensis* is distinguishable from *P. neumanni* call type 1 by having frequency-modulated notes and note groups and longer notes. The call of *P. amharensis* resemble most the call of its closely related *P. cooperi*, although with a higher peak frequency, reflective of the smaller size of the species, and notes grouped within each call while the notes of *P. cooperi* calls are regularly spaced.

#### Remarks.

Neither the holotype designated by Smith et al. (2017), which is a juvenile in poor condition with one missing leg, nor the diagnosis provided in the original description provide characters allowing the distinction between *P. amharensis* and other species of the *P. neumanni* complex. The SVL values given for males and females in Smith et al. (2017a, b) do not correspond to measurements taken on the type series (holotype: juvenile SVL 21.8 mm, paratypes: adult females, SVL 40.2, 43.3, 43.8 mm) and were taken from a summary table (table 4 in Freilich et al. 2014) with no acknowledgement of the original data, and specimens of the type series have evidently not been examined by the authors. The altitudinal range for the species was taken from the same table, but values were rounded in Smith et al. (2017a, b).

### Ptychadena erlangeri

Taxon classificationAnimaliaAnuraPtychadenidae

(Ahl, 1924)

87B773BC-7FF3-5B5B-818D-A5DB58E809FC

Rana erlangeri Ahl, 1924: 4.
Ptychadena erlangeri – Perret 1980: 151–168.
Rana (Ptychadena) erlangeri – Dubois 1981: 233.
Ptychadena (Ptychadena) erlangeri – Dubois 1992: 316.
Ptychadena largeni Perret 1994: 67.

#### Type material.

***Holotype*.** A gravid female (ZMB–26887) collected by C. von Erlanger in December 1900 at lake Abaya, Ethiopia (likely the eastern shore of the lake, 1300 m a.s.l., see remarks below).

#### Material examined.

Except for the type specimen and the type series of *P. largeni*, all the material examined is deposited at ZNHM. One female (15–47) collected by X. Freilich, J. Reyes-Velasco and S. Boissinot south of Assela (7.9068°N, 39.1238°E, 2520 m a.s.l.), one male (15–400) collected by X. Freilich, J. Reyes-Velasco and S. Boissinot on 22 August 2015 north of Fitche (9.7877°N, 38.6974°E, 2821 m a.s.l.), two males (15–417 and 15–420) collected by X. Freilich, J. Reyes-Velasco and S. Boissinot on 26 August 2015 north (6.3844°N, 38.5927°E, 2655 m a.s.l.) and south (6.3256°N, 38.6645°E, 2684 m a.s.l.) of Bore, respectively, one male (16–6) collected by J. Reyes-Velasco and S. Boissinot on 4 July 2016 between Addis Ababa and Ambo, eight males (16–6, 16–10, 16–11, 16–12, 16–14, 16–16, 16–17 and 16–24) collected by J. Reyes-Velasco and S. Boissinot on 5 July 2016 south of Assela (7.7776–7.8431°N, 39.1384–39.1529°E, 2553–2637 m a.s.l.), one female (16–131) and six males (16–99, 16–106, 16–112, 16–113, 16–114 and 16–118) collected by J. Reyes-Velasco and S. Boissinot on 12 July 2016 southeast of Mehal Meda (9.9894–10.3316°N, 39.7452–39.8092°E, 3017–3394 m a.s.l.), one female (16–142) and one male (16–155) collected by J. Reyes-Velasco and S. Boissinot on 13 July 2016 north of Debre Birhan (9.6822–9.6979°N, 39.5505–39.5628°E, 2833–2837 m a.s.l.), one male (16–166) collected by J. Reyes-Velasco and S. Boissinot on 14 July 2016 south of Fitche (9.7502°N, 38.7445°E, 2726 m a.s.l.), one female (SB231) and one male (SB232) collected by S. Goutte and J. Reyes-Velasco on 23 April 2018 south of Gumer (7.9125°N, 38.0644°E, 2831 m a.s.l.), two males (SB552 and SB553) collected by S. Goutte and Y. Bourgeois on 3 July 2018 east of Mehal Meda (10.3247°N, 39.8092°E, 2795 m a.s.l.), one male (SB562) collected by S. Goutte and Y. Bourgeois on 4 July 2018 east of Mehal Meda (10.3247°N, 39.8092°E, 2795 m a.s.l.), three males (SB570, SB571 and SB577) collected by S. Goutte and Y. Bourgeois on 7 July 2018 north of Gebre Guracha (9.8818°N, 38.3660°E, 2558 m a.s.l.), one male (SB615) collected by S. Goutte and Y. Bourgeois on 14 July 2018 near Holeta (9.0692°N, 38.5214°E, 2397 m a.s.l.), *P. largeni* male holotype (MHNG–2513.31), *P. largeni* male paratypes (MHNG–2513.38, MHNG–2513.42, MHNG–2513.44, MHNG–2513.45, MHNG–2513.49, MHNG–2513.52), *P. largeni* female paratypes (MHNG–2513.56, MHNG–2513.57, MHNG–2513.58, MHNG–2513.59 and MHNG–2513.60) collected by M. Largen on 12 June 1977 in Addis Ababa, Shewa (Shoa) (9.03°N, 38.75°E, 2500 m a.s.l.).

#### Diagnosis.

**Figure 11.**
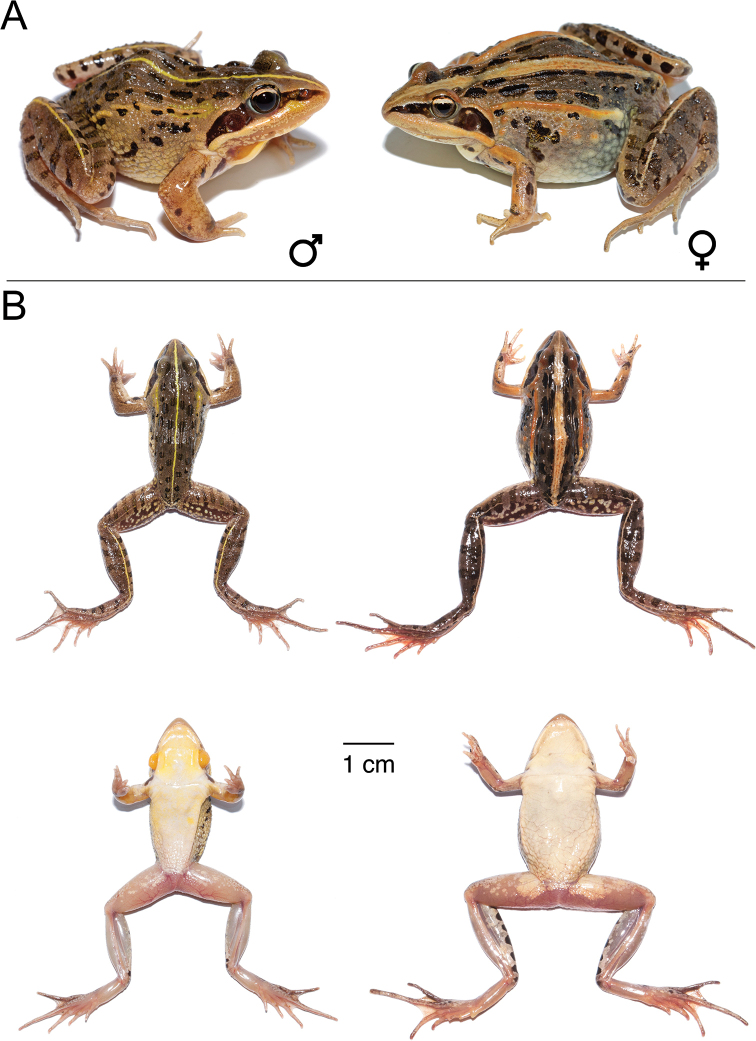
*Ptychadena erlangeri***A** live male (SB571; left) and female (SB548, right) **B** dorsal and ventral views of the same individuals (male left, female right) after euthanasia and before fixation.

A medium-sized (male (35) SVL 34.1 ± 2.5 mm, female (10) SVL 34.6 ± 1.7 mm) member of the *erlangeri* species group (Fig. 11). *Ptychadena erlangeri* differs from other members of the *P. neumanni* complex by the following combination of characters: (1) relatively short hind limbs (male TL/SVL 0.53 ± 0.04, female TL/SVL 0.54 ± 0.05), (2) tympanum translucent, without any light bar or blotch, (3) vocal sacs light in color, from cream to light grey, very rarely with a bit of grey, (4) adult males with robust forelimbs, (5) adult males often covered in small warts.

#### Comparison.

Species very variable in body size across its range, but always smaller than *P. cooperi*, *P. goweri* and larger than *P. nana* and *P. robeensis* sp. nov. *Ptychadena erlangeri* can be distinguished from *P. beka* sp. nov., *P. delphina* sp. nov., *P. doro* sp. nov., *P. amharensis*, *P. neumanni*, and *P. robeensis* sp. nov. by the absence of light bar or blotch on the tympanum. Furthermore, adult males can be distinguished by their cream or light-yellow vocal sac from *P. beka* sp. nov., *P. neumanni*, *P. robeensis* sp. nov., *P. doro* sp. nov., and *P. delphina* sp. nov. *Ptychadena erlangeri* has a shorter snout than *P. amharensis*, *P. harenna*, *P. beka* sp. nov., *P. delphina* sp. nov., *P. goweri*, *P. cooperi*, and *P. doro* sp. nov. Finally, *P. erlangeri* can be distinguished from *P. levenorum* by longer eye-nostril and inter-orbital distances and longer hind limbs.

#### Description of the holotype.

**Figure 12.**
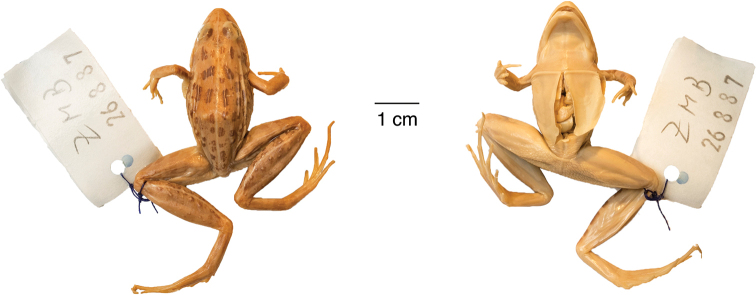
Holotype of *Ptychadena erlangeri*. Dorsal and ventral views of the female holotype (ZMB–26887).

Medium sized (SVL 37.6 mm), slender, gravid female with long hind limbs (TL/SVL 0.63, Fig. 12, Suppl. material 3: Table S1). Head longer than wide (HW/HL 0.94). Snout pointed, projecting beyond the lower jaw. Interorbital distance 0.58 × eye diameter. Nostril half-way between the tip of the snout and the eye. Internarial distance 1.2 × interorbital distance. Tympanum 0.61 × eye diameter. Finger tips not expanded but rounded, with moderate subarticular tubercles. Finger formula: I<II<IV<III. Hand free of webbing. Hindlimbs elongated, with tibia length 0.63 × snout-vent length. Rounded white warts irregularly spread on the dorsal side of tibia. Foot as long as thigh and almost as long as tibia (FL/TL 0.98). Toe tips rounded. Subarticular tubercles small and round. Inner metatarsal tubercle present, external absent. Toe formula: I<II<III<V<IV (right foot) and I<II<VIII<IV (left foot). Foot webbing formula (toe internal/external sides, number of phalanges webbed): Ie(1), IIi/e(1–2), IIIi/e(2–2), IVi/e(2–2), Vi(2). Two light, continuous lateral ridges, four interrupted dorsal ridges. No vertebral nor sacral ridges. Note that we did not see the nasal ridges that Perret (1980) noted as the single major diagnostic character for the species.

#### Coloration of the holotype in preservative.

Although the holotype is in overall very good condition, the coloration seems to have faded away with time and some dark markings noted by Ahl (1924) in the original description and Perret (1980) are now barely visible or have vanished altogether. Dorsal ground color light brown with irregular oval dark brown blotches symmetrically distributed on the dorsolateral ridges. Thin, cream vertebral stripe from the tip of the snout to the vent. Dark brown canthal stripe from the tip of the snout to the back of the jaw. Upper lip and flanks cream. The dark markings on the upper and lower lips noted by Ahl (1924) are no longer visible. Throat, ventrum, ventral side of the thighs, and tibias uniformly cream without any melanization. A thin, cream longitudinal stripe on the tibias and half of the thighs. Dark brown bars on the tibias and brown markings on the thighs.

#### Variations.

*Ptychadena erlangeri* shows important variations in body size, morphology, and coloration across its large distribution range. South of the GRV, individuals of *P. erlangeri* are generally larger (male (9) SVL 36.8 ± 1.9 mm, female (1) SVL 35.6) than north of the GRV (male (17) SVL 33.5 ± 1.9 mm, female (3) SVL 35.4 ± 1.2). Morphometric variations are summarized in Table 1.

In life, dorsum coloration varies from dark brown to grey-brown, olive and golden, sometimes with irregular green blotches. All specimens examined had small dark brown or black blotches distributed symmetrically on the dorsal ridges. A few additional dark brown or black blotches are found in the anterior part of the flanks. A vertebral stripe is always present, either thin or wide and may be white, cream, or green.

Iris bicolored, the upper third silver and lower two thirds dark brown. Upper and lower jaws featuring irregular light grey or brown markings but no barring. All specimens examined featured a dark brown canthal stripe from the tip of the snout to the back of the upper lip, with the tympanum uniformly colored and lacking any light stripe of blotch. A thin cream longitudinal stripe on the tibia extends to a fifth to the whole length of the thigh. Tibias, thighs, and feet more or less regularly barred with dark brown. Thighs posteriorly marbled with dark brown and yellowish brown. Ventrum and throat uniformly cream to yellow. Vocal sacs cream, yellowish or light grey, very rarely with a bit of grey. Small warts over the body and forelimbs present in 80% of adult males examined.

#### Habitat, distribution, and natural history.

*Ptychadena erlangeri* has a wide distribution range extending on both sides of the Great Rift Valley (6.23–10.33°N, 38.06–39.81°E). It is restricted to higher altitudes, from 2387 m to 3394 m a.s.l. (based on 156 barcoded individuals). West of the Great Rift Valley, its range is limited by the Blue Nile valley and specimens have been found just north of Gebre Guracha. Its northernmost locality is Mehal Meda. Southeast of the GRV, populations have been found near Assela, Kofele and Irba Moda. A few individuals have been found in the southwest, between Tippi and Gech’a, and at lower elevation than any other population (7.4512°N, 35.3992°E, 2270 m a.s.l.). GPS coordinates for all examined specimens are given in Suppl. material 3: Table S1. *Ptychadena erlangeri* is found in syntopy with *P. beka* sp. nov. at the lower end of its altitudinal range, notably near Fitche, Holeta, between Ambo and Wonchi, and possibly near Assela. It is also found in sympatry with *P. cooperi* across its range.

Males are found calling in shallow puddles, flooded grasslands, or agricultural fields. Males can be found vocalizing very close to one another, sometimes in important numbers. Calling activity depends on rainfall and is highest during rainy months. Calling usually starts after 22:00, and sometimes as late as 02:00 in dry weather, and ceases before dawn. Numerous, small bicolored eggs are laid in the same water body.

#### Advertisement call.

The call of *Ptychadena erlangeri* (5 males, 226 calls) is composed of a single pulsed note of 290 ± 35 ms in duration, containing 46.5 ± 1.6 pulses. Within calls, pulses are grouped, with 4.1 ± 2.5 pulses per group and the first pulse typically notably lower in amplitude than the other pulses of the pulse group. Amplitude of the call increases gradually during most of the call, peaking at 230 ± 14 ms, and decreases abruptly afterwards. Call dominant frequency is 2343 ± 454 Hz.

The call of *P. erlangeri* can be differentiated from those of *P. cooperi*, *P. amharensis*, *P. doro* sp. nov. and *P. neumanni* (type A and B) by the distinguishable pulses composing the calls. It is also distinct from the calls of *P. delphina* sp. nov. and *P. robeensis* sp. nov. by its short inter-pulse intervals. The call of *P. erlangeri* differs from the calls of *P. delphina* sp. nov., *P. robeensis* sp. nov., *P. levenorum*, *P. beka* sp. nov. and *P. goweri* by its short duration. Finally, the call of *P. erlangeri* differs from the call of *P. nana* by its longer duration and lower pulse rate.

#### Remarks.

Confusion and difficulty to distinguish *P. erlangeri* from *P. neumanni* arose from the original descriptions of the species themselves, both published by Ahl in 1924 in the same article. The original description of *P. neumanni*, based on 35 syntypes, most likely contained three distinct species (Perret 1980), while *P. erlangeri* was described based on a single gravid female. Perret (1980) restricted *P. neumanni* to three male syntypes. Comparing the two closely related species was thus rendered near impossible due to the low sample size and the fact that each species was represented by specimens of different sexes. Adding to the confusion, in 1994, Perret described *Ptychadena largeni* from 30 specimens from Addis Ababa, Shewa (Shoa) sent to him by Largen as *Ptychadena erlangeri*. Largen (1997) then considered that the morphological diagnostic characters found by Perret in the 30 specimens were due to conservation artefacts as these individuals were fixed in alcohol rather than in formalin, and subsequently synonymized *P. largeni* with *P. neumanni* in 2001, even though he had originally considered those specimens to belong to *P. erlangeri*. Recently, molecular phylogenetic analyses grouped the holotype of *P. largeni* with that of *P. erlangeri* (Reyes-Velasco et al. in review).

Largen (2001) thus included a population of *P. erlangeri* (*P. largeni*) in *P. neumanni*, along with specimens from Debre Markos, Gondar, the Bale Mountains, etc. and included specimens from the Harenna forest and Debre Markos in *P. erlangeri*. The two groupings thus comprised specimens of several species, many of which placed in both groups (Fig. 1). As a result, Largen (2001) failed to give satisfactory diagnostic characters distinguishing *P. erlangeri* from *P. neumanni* as all the given characters largely overlapped, and later authors relied heavily on the distribution ranges given by Largen to assign species names to the populations they sampled. Notably, the photos presented in Largen and Spawls (2010) for *P. erlangeri* is in fact most likely a *P. goweri* (Yadot River, close to Dolo Mena described as the “large” form occurring in Bale Mountain by Largen 1997), and the specimens presented as *P. neumanni* are in fact *P. erlangeri*.

### Ptychadena levenorum

Taxon classificationAnimaliaAnuraPtychadenidae

Smith, Noonan & Colston, 2017

18DEDED7-3454-56FA-A395-B2D2793E692D

#### Type material.

***Holotype*.** Male, TJC219, collected in Katcha, Bale Moutains National Park, Ethiopia (6.7165°N, 39.7248°E, 2326 m a.s.l.) by T. Colston on 8 December 2012. ***Paratypes*.** XF923 and XF927, collected by X. Freilich and S. Boissinot on the 9 August 2011 west of Dinsho (7.1112°N, 39.7430°E, 3024 m a.s.l.). All type specimens and material examined are deposited at ZNHM.

#### Material examined.

Beside the male holotype (TJC219) we examined one female (15–155) and one male (15–158) collected by X. Freilich, J. Reyes-Velasco, and S. Boissinot on 9 August 2015 east of Dinsho (7.1061°N, 39.8181°E, 3058 m a.s.l.), one female (15–482) collected by X. Freilich, J. Reyes-Velasco and S. Boissinot on 3 October 2015 south of Dodola (6.8465°N, 39.1933°E, 3404 m a.s.l.), one female (16–89) and one male (16–90) collected by J. Reyes-Velasco and S. Boissinot on 10 July 2016 east of Dinsho (7.1065°N, 39.8184°E, 3065 m a.s.l.), one male (SB43) collected by S. Goutte and J. Reyes-Velasco on 6 April 2018 south of Dodola (6.8632°N, 39.1948°E, 3260 m a.s.l.), two males (SB66 and SB67) collected by S. Goutte and J. Reyes-Velasco on 8 April 2018 south of Dinsho, one female (SB85) and two males (SB90 and SB94) collected by S. Goutte and J. Reyes-Velasco on 9 April 2018 east of Dinsho.

#### Diagnosis.

**Figure 13.**
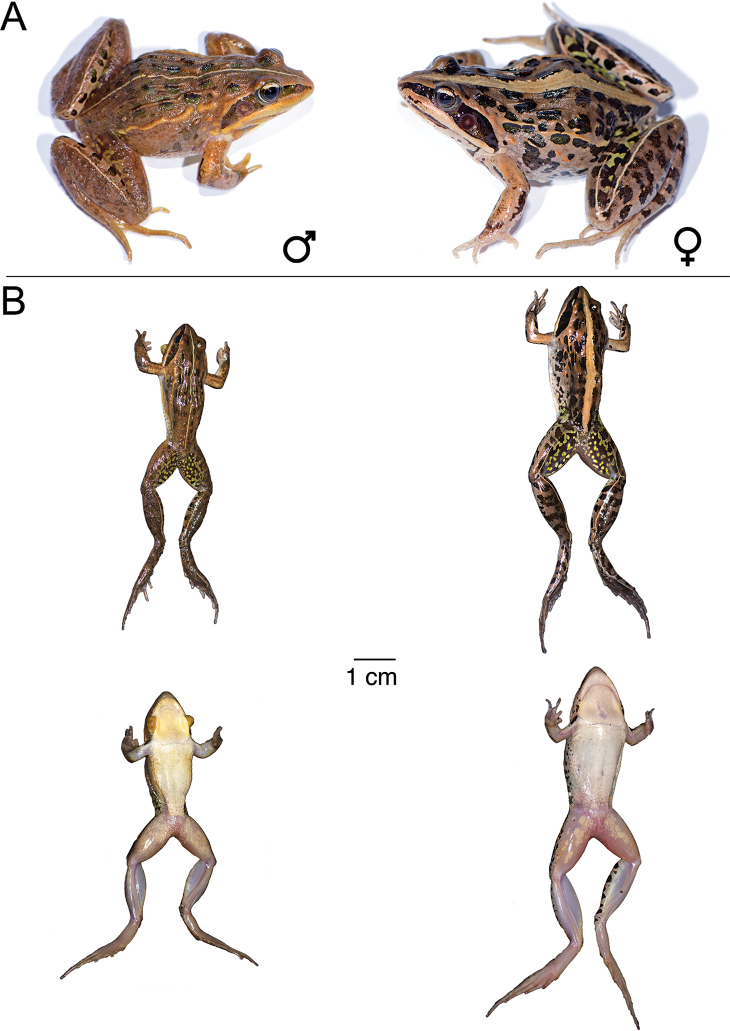
*Ptychadena levenorum***A** live male (SB90; left) and female (SB85, right) **B** dorsal and ventral views of the same individuals (male left, female right) after euthanasia and before fixation.

A small species (male (8) SVL 34.3 ± 2.3 mm, female (4) SVL 37.0 ± 2.5 mm) of the *erlangeri* species group (Fig. 13) with variable coloration, distinguished by the following combination of characters: (1) short hindlimbs (male TL/SVL 0.49 ± 0.06, female TL/SVL 0.51 ± 0.04), (2) head as long as wide, (3) snout short (male SN/SVL 0.13 ± 0.02, female SN/SVL 0.13 ± 0.01), (4) vocal sacs cream, light grey, or bicolored cream and grey.

#### Comparison.

Smaller than *P. goweri*, *P. delphina* sp. nov., and *P. cooperi* and larger than *P. nana* and *P. robeensis* sp. nov. Snout shorter than all species of the *P. neumanni* complex except for *P. nana*, *P. robeensis* sp. nov., and *P. erlangeri*. Inter-orbital and eye-nostril distances shorter than *P. erlangeri*. Thigh and tibia of *P. levenorum* are shorter than those *P. erlangeri*, *P. doro* sp. nov., *P. harenna*, *P. goweri*, and *P. neumanni*, and longer than those of *P. nana* and *P. robeensis* sp. nov. Shorter feet than *P. neumanni* and *P. doro* sp. nov. Shorter hands than *P. amharensis*. Tympanum larger than in *P. nana* and *P. robeensis* sp. nov.

#### Description of the holotype.

**Figure 14.**
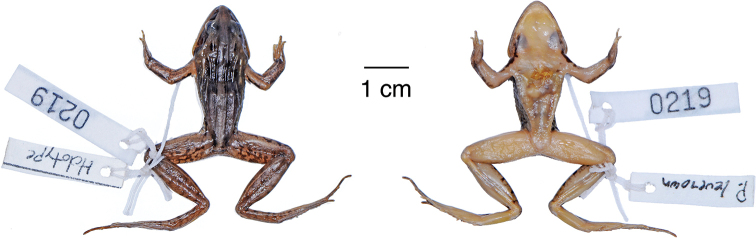
Holotype of *Ptychadena levenorum*. Dorsal and ventral views of the male holotype (TJC219).

Small sized (SVL 33.3 mm) male (Fig. 14). Head longer than wide (HW/HL 0.93). Snout slightly rounded, projecting beyond the lower jaw. Interorbital distance shorter than eye diameter (IOD/ED 0.78) and internarial distance (IOD/IND 0.93). Nostril closer to the eye than the tip of the snout. Tympanum 0.72 × eye diameter. Finger tips not expanded but rounded. Finger formula: I<II<IV<III. Hand free of webbing. Hindlimbs moderately long, with tibia length 0.54 × snout-vent length. Thigh shorter than tibia. Foot 1.3 × tibia length. Toe tips rounded. Inner metatarsal tubercle present, outer absent. Toe formula: I<II<V<III<IV. Foot webbing formula (toe internal/external sides, number of phalanges webbed): Ie(1), IIi/e(1–1.5), IIIi/e(1.5–2), IVi/e(2–2), Vi(2). Four discontinuous dorsal ridges and two discontinuous cream lateral ridges.

#### Coloration of the holotype in preservative.

Dorsal ground color brown with few small irregular oval dark brown blotches symmetrically distributed on the dorsolateral ridges. Very faint wide light grey-brown vertebral stripe from the tip of the snout to the vent. One faint interrupted dorsolateral cream ridge on each side. Dark brown canthal stripe from the tip of the snout to the back of the jaw. Upper lip cream dusted with brown and with irregular dark brown markings. Flanks cream with a few large and irregular dark brown spots. Throat, ventrum, ventral side of the thighs and tibias uniformly cream without any melanization, except for a couple of small irregular brown blotches on the upper ventrum. Barely visible dark brown bars on the tibias and feet. Irregular dark brown markings on the thighs, arms and forearms. Back of the thighs light brown marbled dark brown. Tympanum uniformly dark brown. Vocal sacs cream with some grey markings.

#### Variations.

*Ptychadena levenorum* shows important variations in coloration. In life, dorsum coloration varies from light yellowish brown to dark reddish brown, dark brown and bright green. All specimens examined had black blotches distributed symmetrically on the dorsal ridges, and for some of them the blotches were black with a green iridescence. A few additional dark brown or black blotches are found in the anterior part of the flanks. A vertebral stripe is always present, either thin, medium, or wide and may be cream, yellow, pale brown or bright green.

Iris bicolored, the upper third silver to golden and lower two thirds dark brown. Upper and lower jaws white to golden with few irregular light grey or brown markings but no barring. All specimens examined featured a dark brown or iridescent green canthal stripe from the tip of the snout to the back of the upper lip. Tympanum dark brown, sometimes with golden-iridescent green undefined blotch or dusting. A thin cream or green longitudinal stripe on the tibia is present in some individuals and may extend to the foot and to part or the entire length of the thigh. Tibias, thighs and feet more or less visibly barred with dark brown. Thighs posteriorly dark brown marbled with yellow. Ventrum and throat uniformly white to light yellow. Vocal sacs cream or light grey, very rarely with a bit of grey anteriorly. Small warts over the body and forelimbs present in 40% of adult males examined.

#### Habitat, distribution, and natural history.

The relatively restricted distribution range of *Ptychadena levenorum* extends on both the northern and southern sides of the Sanetti plateau (6.72–9.42°N, 38.66–39.82°E), although there is no record of its presence on the plateau itself. This species occurs mostly at elevation higher than 3000 m a.s.l. (3015 to 3404 m a.s.l.), except for the population living at the type locality Katcha (6.72°N, 39.72°E, 2410 m a.s.l.). The westernmost population was found south of Dodola, near Garamba (6.86°N, 39.19°E, 3260 m a.s.l.).

Individuals have been found, generally in small numbers, calling at night from shallow puddles in grassy meadows or forest clearings. Within the genus, this species is found in sympatry with *Ptychadena cooperi* and *P. robeensis* sp. nov. in the northern part of its range, and *Ptychadena harenna* and *P. goweri* at the type locality. Genetic analyses have shown that *P. levenorum* and *P. robeensis sp. nov*. hybridize where their ranges overlap.

#### Advertisement call.

The call of *Ptychadena levenorum* (2 males, 30 calls) is composed of a single pulsed note of 465 ± 98 ms in duration, containing 23.5 ± 4.3 pulses. Within a call, pulses are grouped, with 4.1 ± 1.5 pulses per group and sometimes with a lower-amplitude single pulse between pulse groups. The duration of inter-pulse intervals within pulse groups is 12 ± 2 ms. Call peak frequency is 2223 ± 90 Hz.

The call of *P. levenorum* differs from those of *P. cooperi*, *P. amharensis*, *P. doro* sp. nov. and *P. neumanni* (type A and B) by the distinguishable pulses composing the calls. Grouped pulses and short inter-pulses intervals distinguish the call of *P. levenorum* from those of *P. robeensis* sp. nov. and *P. delphina* sp. nov. The call of *P. levenorum* can be further distinguished from those of *P. nana* and *P. erlangeri* by a longer duration and from *P. goweri* by a shorter duration and fewer pulses.

### Ptychadena nana

Taxon classificationAnimaliaAnuraPtychadenidae

Perret, 1980

A4613A6C-39D7-55EF-8134-4DEF810341F6

Ptychadena nana Perret, 1980: 160.
Rana (Ptychadena) nana – Dubois 1981: 233.
Ptychadena (Ptychadena) nana – Dubois 1992: 316.

#### Type material.

***Holotype*.** A female (ZMB–26878 H) collected by O. Neumann and C. von Erlanger between June and mid-August 1900 (see Neumann 1902) in “Somaliland”, which corresponds to Didda, East Arussi plateau, Ethiopia, 2000–3000 m a.s.l. according to Largen and Perret (Largen 1975, 1977; Perret 1980) [Coordinates estimated by Largen (1997): 7.83°N, 39.50°E]. ***Paratypes*.** From the 20 paratypes included in the original description (Perret 1980), 16 specimens (ZMB–26877, ZMB–57185 up to and including ZMB–57199) collected by O. Neumann and C. von Erlanger at the same time and location are in the collections of the Berlin Museum and four have either been lost or placed in another collection.

#### Material examined.

Except for the type series, all the material examined is deposited at ZNHM. Beside the female holotype (ZMB–26878 H), we examined four female paratypes (ZMB–57189, ZMB–57190, ZMB–57193, ZMB–57199) and four male paratypes (ZMB–26877, ZMB–57191, ZMB–57192, ZMB–57195) collected by C. von Erlanger and O. Neumann in 1900 likely between June and mid-August in Didda, East Arussi plateau, one female (SB488) and two males (SB486, SB487) collected by S. Goutte and Y. Bourgeois on 26 June 2018 west of Ch’ange (8.1303°N, 39.3985°E, 2357 m a.s.l.), one female (SB493) and two males (SB494, SB495) collected by S. Goutte and Y. Bourgeois on 27 June 2018 between Robé and Sedika (7.7307°N, 39.7133°E, 2377 m a.s.l.), five females (SB508, SB510, SB512, SB515, SB516) and four males (SB523, SB524, SB525, SB526) collected by S. Goutte and Y. Bourgeois on 29 June 2018 southeast of Ch’ange (8.1086°N, 39.4486°E, 2573 m a.s.l.).

#### Diagnosis.

**Figure 15.**
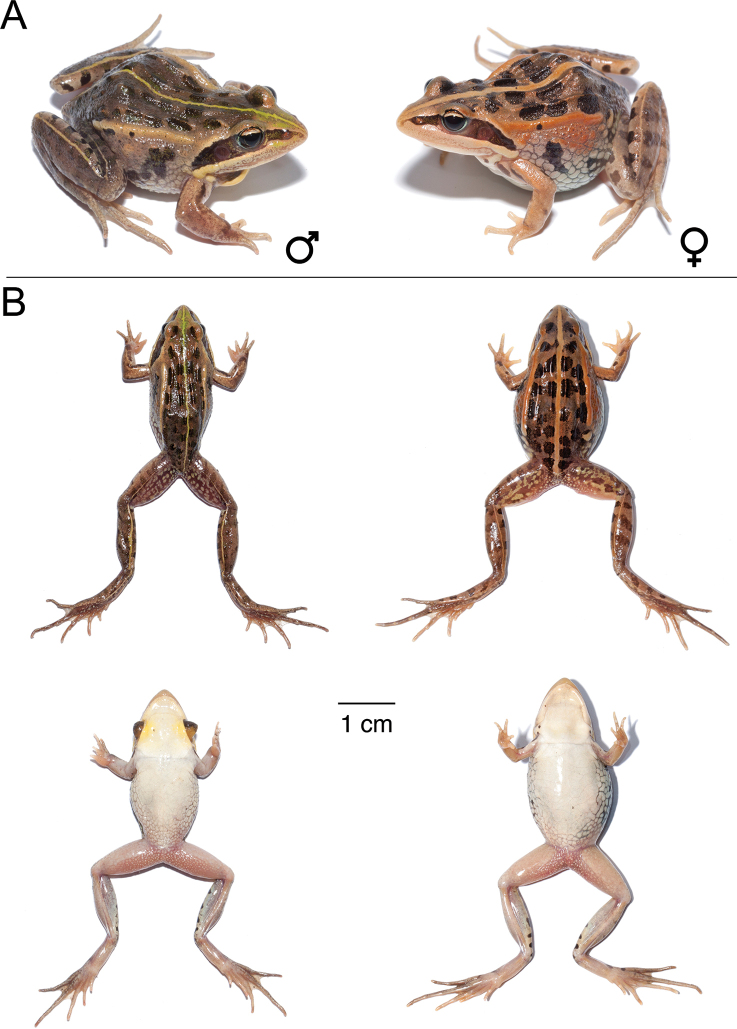
*Ptychadena nana***A** live male (SB494; left) and female (SB488, right) **B** dorsal and ventral views of the same individuals (male left, female right) after euthanasia and before fixation.

The smallest species (male (12) SVL 28.3 ± 3.3 mm, female (12) SVL 29.6 ± 3.2 mm) of the *P. neumanni* complex (Fig. 15), distinguished by the following combination of characters: (1) short hind limbs (male TL/SVL 0.45 ± 0.03, female TL/SVL 0.44 ± 0.03), (2) short hands (HAL/SVL 0.20 ± 0.02), (3) short feet (male FL/SVL 0.49 ± 0.04, female FL/SVL 0.48 ± 0.04), (4) reduced foot webbing, (5) no stripe or blotch on the tympanum, (6) adult males almost always covered in small warts during the breeding season.

#### Comparison.

Distinguished from all Ethiopian *Ptychadena*, except for *P. robeensis* sp. nov., by a considerably smaller size. Very similar to *P. robeensis* sp. nov., *P. nana* has relatively shorter hand, shorter head, a shorter eye-tympanum distance and shorter feet than *P. robeensis* sp. nov. The tympanum lacks any light marking, as opposed to *P. robeensis* sp. nov. Small warts almost always present in males, whereas they are absent in *P. robeensis* sp. nov.

#### Description of the holotype.

**Figure 16.**
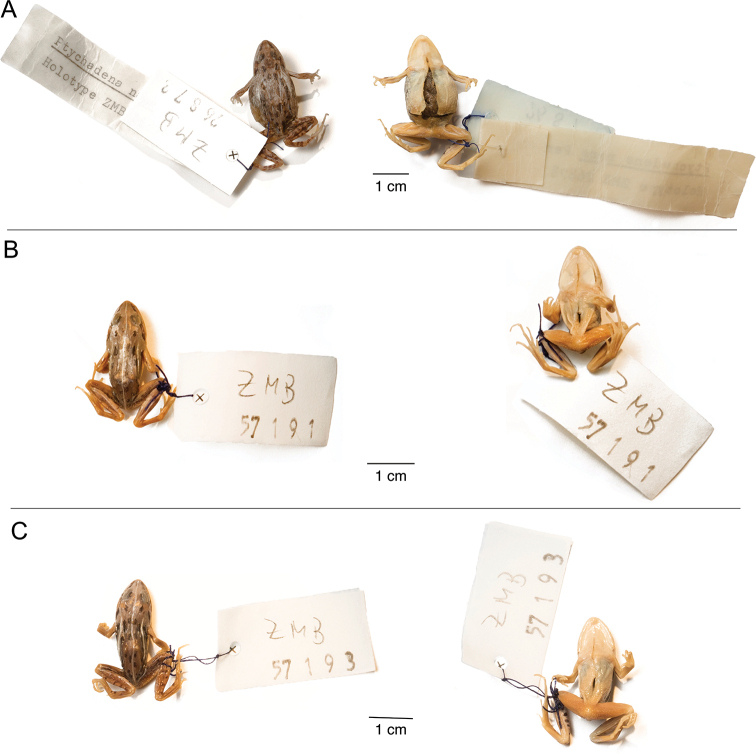
Type specimens of *Ptychadena nana*. Dorsal and ventral views of three specimens from the type series **A** female holotype (ZMB–26878 H) **B** male paratype (ZMB–57191) **C** female paratype (ZMB–57193).

Small sized (SVL 26.9 mm) gravid female with short hind limbs (TL/SVL 0.43, Fig. 16, Suppl. material 3: Table S1). Head longer than wide (HW/HL 0.95). Snout rounded, slightly projecting beyond the lower jaw. Interorbital distance 0.58 × eye diameter. Nostril half-way between the tip of the snout and the eye. Internarial distance 1.4 × interorbital distance. Tympanum 0.65 × eye diameter. Finger tips not expanded but rounded. Finger formula: III<IV<III. Hand free of webbing. Hindlimbs short, with tibia length 0.42 × snout-vent length. Foot 1.2 × tibia length and 1.3 thigh length. Toe tips rounded. Outer metatarsal tubercle absent. Toe formula: I<II<V<III<IV. Foot webbing formula (toe internal/external sides, number of phalanges webbed): Ie(1), IIi/e(1–1), IIIi/e(1–2), IVi/e(2–2), Vi(2). Ridges hardly distinguishable due to the state of preservation of the specimen.

#### Coloration of the holotype in preservative.

Dorsal ground color brown with irregular oval dark brown blotches symmetrically distributed, presumably on the dorsolateral ridges. One dark brown blotch on each upper eyelid. No vertebral stripe. One fainted dorsolateral cream ridge on each side. Dark brown canthal stripe from the tip of the snout to the back of the jaw. Upper lip cream. Flanks brown to dark brown with numerous cream spots which merge ventrally. Throat, ventrum, ventral side of tibias uniformly cream without any melanization. Ventral side of the thighs light brown. Dark brown bars on the thighs, tibias and feet. Back of the thighs brown marbled with cream. Dark brown irregular markings on the arms and forearms. No stripe or blotch on the tympanum.

#### Variations.

As the other members of the *Ptychadena erlangeri* species group, *Ptychadena nana* shows color polymorphism. In life, dorsum coloration varies from light grey to sand and brown. The dark blotches disposed more or less symmetrically on the dorsum vary in size and number, sometimes covering more than half the dorsum. Their colors vary from olive-brown, with some green iridescence, to black. One or several blotches of the same color, sometimes fused into a large irregular shape, were present on the anterior part of the flank in all examined specimens. The vertebral stripe, when present, may be thin, medium or wide and cream, light brown or bright green. Some individuals lack any vertebral stripe.

Iris bicolored, the upper third silver to golden and lower two thirds brown to dark brown. Upper and lower jaws cream, golden or light brown, often with irregular light grey or light brown markings but no barring. All specimens examined featured a dark brown canthal stripe from the tip of the snout to the back of the upper lip, sometimes dusted with small green spots. Tympanum uniformly colored and lacking any light stripe of blotch. A thin cream or light green longitudinal stripe on the tibia extending the complete length of the thigh is present in some individuals. Barring on the tibias, thighs, and feet may be very distinct or almost completely absent. Thighs posteriorly marbled with dark brown and yellow or cream. Ventrum uniformly white. Throat white in females and pale yellow to deep yellow in adult males. Vocal sacs bicolored grey anteriorly and cream posteriorly, rarely completely grey. Small warts over the body and forelimbs present in 90% of adult males examined.

#### Habitat, distribution, and natural history.

*Ptychadena nana* is found on the eastern half of the Arussi plateau (7.64–8.30°N, 39.39–39.87°E, Oromia, Ethiopia from 2380 to 2850 m a.s.l. In the south, its range is limited by the Shebelle River, while in the northeast it is bordered by the Great Rift Valley. The westernmost individuals were found around four kilometers west of Ch’ange. The steep terrain just east of Huruta may serve as geographic barrier for dispersal in this species, as it has not been found further southeast. In the southwest end of its range, *P. nana* has been found just north of Dibe, but presumably occurs all the way down to Barbari as there is no physical barrier that would prevent its dispersal on this side of the plateau.

Males are found calling at night in large numbers from shallow roadside puddles, usually overgrown by grass. Within the genus, *P. nana* is found in sympatry with *Ptychadena cooperi*.

#### Advertisement call.

The call of *Ptychadena nana* (5 males, 56 calls) is composed of a single pulsed note of 205 ± 47 ms in duration, containing 16.9 ± 2.8 pulses. Inter-pulses intervals are very short (9 ± 2 ms) and irregular, forming few irregular pulse groups and resulting in a high pulse rate (84 ± 10.2 pulses s^-1^). Call peak frequency is 2461 ± 26 Hz.

The call of *P. nana* can be differentiated from those of *P. cooperi*, *P. amharensis*, *P. doro* sp. nov. and *P. neumanni* (types A and B) by the distinguishable pulses composing the calls. It is also distinct form the calls of *P. delphina* sp. nov. and *P. robeensis* sp. nov. by its short inter-pulse intervals. Finally, the call of *P. nana* differs from the calls of *P. delphina* sp. nov., *P. robeensis* sp. nov., *P. erlangeri*, *P. levenorum*, *P. beka* sp. nov., and *P. goweri* by its short duration and high pulse rate.

### Ptychadena robeensis sp. nov.

Taxon classificationAnimaliaAnuraPtychadenidae

45EEF9BC-D051-5803-A1CF-764921588C18

http://zoobank.org/C43EB165-5CC3-4489-8B07-AACA17E30367

#### Type material.

***Holotype*.** Adult male (SB81) collected on 9 April 2018 by S. Goutte and J. Reyes-Velasco between Robe and Ali (7.2111°N, 39.9672°E, 2374 m a.s.l.). ***Paratypes*.** Three males (15–139, 15–140 and 15–147) and one female (15–138) collected on 8 August 2015 northwest of Robe (7.1720°N, 39.9722°E; 2431 m a.s.l.), one male (15–163) collected on 9 August 2015 east of Dinsho (7.1061°N, 39.8182°E; 3058 m a.s.l.), one male (16–92) and one female (16–77) collected by J. Reyes-Velasco and S. Boissinot 10 July 2016 east of Dinsho (7.1065°N, 39.8184°E, 3065 m a.s.l.), one male (SB65) collected by J. Reyes-Velasco and S. Goutte on 8 April 2018 south of Dinsho (7.0915°N, 39.7834°N, 3079 m a.s.l.), three males (SB82, SB83, SB84) collected by J. Reyes-Velasco and S. Goutte on 9 April 2019 between Robe and Ali (7.2111°N, 39.9672°E, 2374 m a.s.l.), three males (SB88, SB92, SB93) and two females (SB87, SB89) collected by J. Reyes-Velasco and S. Goutte on 9 April 2019 east of Dinsho (7.1056°N, 39.8168°E, 3015 m a.s.l.). All specimens are deposited at ZNHM.

#### Diagnosis.

**Figure 17.**
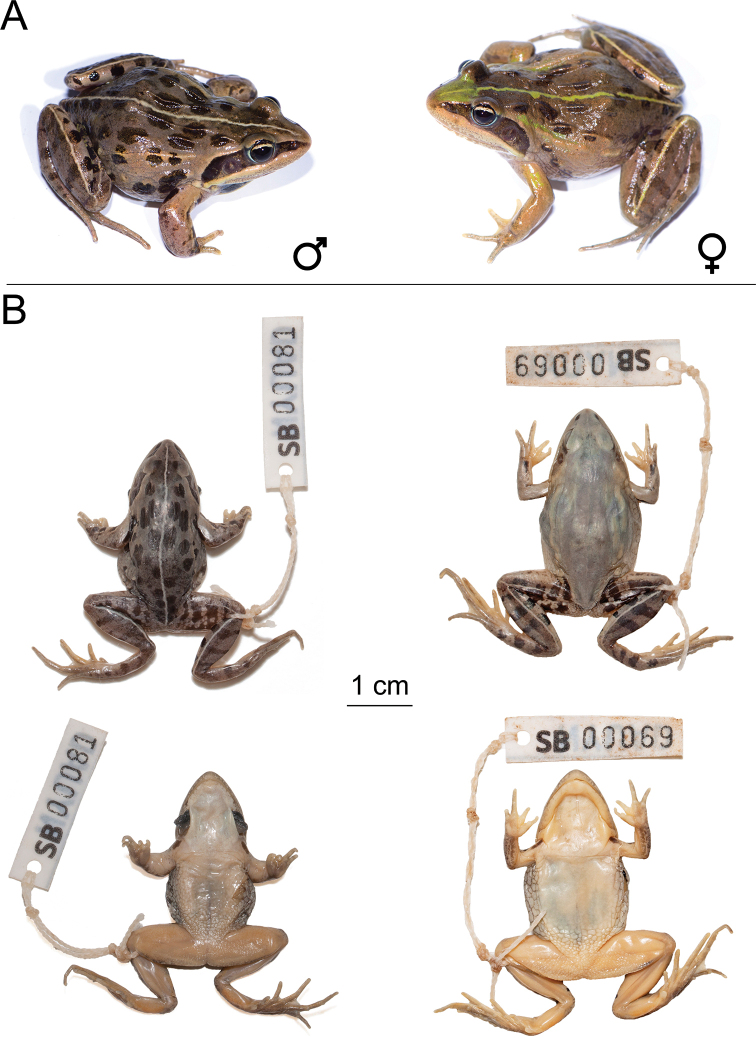
*Ptychadena robeensis* sp. nov. **A** live male holotype (SB81; left) and female paratype (SB89, right) **B** dorsal and ventral views of the male holotype (SB81; left) and a female (SB69; right) after fixation. The female SB69, green in life, lacks any melanization on the dorsum, a rare phenotype that has been encountered in a few individuals only.

Small species (male (13) SVL 30.3 ± 0.7 mm, female (4) SVL 31.6 ± 2.9 mm) of the *erlangeri* species group (Fig. 17), distinguishable from other species of the *P. neumanni* complex by the following combination of characters: (1) short hind limbs (male TL/SVL 0.45 ± 0.0.03, female TL/SVL 0.44 ± 0.02), (2) short forearms (male FLL/SVL 0.18 ± 0.02, female FLL/SVL 0.18 ± 0.00), (3) short feet (male FL/SVL 0.5 ± 0.03, female FL/SVL 0.49 ± 0.05), (4) reduced foot webbing, (5) a vertical cream or golden stripe or blotch on the tympanum (sometimes faint), (6) warts absent in adult males during the breeding season.

#### Comparison.

Distinguished from all Ethiopian *Ptychadena*, except for *P. nana*, by a considerably smaller size. Compared to *P. nana*, it has relatively longer hands, greater eye-tympanum distance and longer feet. *Ptychadena robeensis* sp. nov. also presents a more or less distinct cream or golden blotch on the tympanum, which is absent in *P. nana*. Finally, the bodies of adult male *P. robeensis* sp. nov. are not covered in warts as opposed to *P. nana*.

#### Description of the holotype.

Small sized (SVL 29.3 mm) male with short hind limbs (TL/SVL 0.45, Fig. 17, Suppl. material 3: Table S1). Head longer than wide (HW/HL 0.96). Snout slightly rounded, projecting beyond the lower jaw. Interorbital distance equal to the eye diameter and to the internarial distance. Nostril half-way between the tip of the snout and the eye. Tympanum 0.77 × eye diameter. Finger tips not expanded but rounded. Finger formula: I<II<IV<III. Hand free of webbing. Hindlimbs short, with tibia length 0.45 × snout-vent length. Tibia and thigh lengths equal. Foot 1.3 × tibia length. Toe tips rounded. Inner metatarsal tubercle present, outer absent. Toe formula: I<II<V<III<IV. Foot webbing formula (toe internal/external sides, number of phalanges webbed): Ie(1), IIi/e(1–1), IIIi/e(1–1), IVi/e(1–1), Vi(1). Four continuous dorsal ridges and one faint cream lateral ridge, interrupted on one side and continuous on the other.

#### Coloration of the holotype in life.

Dorsal ground color grey with a few, elongated dark brown blotches symmetrically distributed on the dorsolateral ridges. Thin cream stripe on the dorsum from the tip of the snout to the vent, on the foot, tibia, and half of the thigh. Dark olive brown canthal stripe from the tip of the snout to the back of the jaw. Small cream blotch on the otherwise dark brown tympanum. Upper and lower lip cream to light brown towards the tip of the snout with irregular small brown markings.

Iris bicolored, with upper third light cream, and the lower two thirds dark brown with golden freckles. Irregular dark brown blotches on the flanks. Ventrum cream, reticulated with light brown on its lower part. Throat light yellow. Two small symmetrical dark brown blotches on the antero-ventral side of the shoulders. Hind limbs brown with dark brown bars over the thighs, tibias and feet. A few very small round white dots around the groin. Back of the thighs dark brown irregularly molted with yellow. Vocal sacs dark grey anteriorly and light grey posteriorly.

#### Coloration of the holotype in preservative.

Dorsal ground color grey with a few large oval black blotches symmetrically distributed on the dorsolateral ridges. Thin cream vertebral stripe from the tip of the snout to the vent. Dark brown canthal stripe from the tip of the snout to the back of the jaw. Faint vertical stripe on the otherwise brown tympanum. Upper lip and lower lips cream dusted with light grey. Flanks light with a few irregular small dark brown blotches. Throat, ventrum, ventral side of the thighs and tibias uniformly cream. Two symmetrical brown blotches on the ventral side of the shoulders. Faint, thin longitudinal stripe on the tibia, foot and half of the thigh. Foot, tibia and thigh barred with brown. Back of thighs molted light grey and brown. Vocal sacs dark grey anteriorly and light grey posteriorly. Nuptial pads cream.

#### Variations.

As the other members of the *erlangeri* species group, *Ptychadena robeensis* sp. nov. shows color polymorphism. In life, dorsum coloration varies from grey-brown to reddish or yellowish brown, or bright lime green. The dark blotches disposed more or less symmetrically on the dorsum vary in size and number and are either dark brown or black. A few individuals completely lack melanization on the dorsum, resulting in an almost uniform light brown or bright lime green coloration. The limbs of these individuals, however, have melanization patterns comparable to other individuals.

One or a few dark blotches is present on the anterior part of the flank of some individuals.

The vertebral stripe, when present, may be thin or wide and cream, sand, yellow or bright lime green. Wide stripes may be doubled with a thin, lighter line. Some individuals lack any vertebral stripe and individuals lacking dorsal melanization generally have a barely visible thin light vertebral stripe.

Iris bicolored, the upper third cream, silver or golden and lower two thirds brown to dark brown. Upper and lower jaws cream, golden or light green, often with irregular light grey or light brown markings but no barring. Most individuals feature a dark brown canthal stripe from the tip of the snout to the back of the upper lip, sometimes dusted with small green spots. Individuals lacking dorsal melanization have a golden canthal stripe covered with small dark grey spots. Interestingly, the nostrils of these individuals are outlined in black. Tympanum dark brown with a more or less defined cream to golden vertical blotch. A thin cream or light green longitudinal stripe on the tibia extending to half or the whole length of the thigh is present in some individuals. Barring on the tibias, thighs and feet may be very distinct or almost completely absent. Thighs posteriorly marbled with dark brown and light brown, yellow or light green. Ventrum uniformly white. Throat white or very pale yellow in females and pale to deep yellow in adult males. Vocal sacs dark grey in 70% of examined specimens, bicolored dark grey anteriorly and light grey posteriorly in some individuals, and rarely light grey. Warts were absent from all adult males examined.

#### Etymology.

The specific name refers to the town of Robe, near the type locality.

#### Habitat, distribution and natural history.

*Ptychadena robeensis* sp. nov. occupies a very small range (7.05–7.23°N, 39.78–39.98°E) around the town of Robe, Bale zone, Oromia, Ethiopia. It is found at elevations ranging from 2375 m to 3120 m a.s.l. The westernmost individuals were found near Dinsho, which is also the highest elevation point of the species range. In the south, *P. robeensis* sp. nov. is found between Robe and Goba, while it is known up to just west of Ali in the north. The species presumably occurs further north on the plateau as elevation and habitat seem rather homogeneous up to the Shebelle River.

Males are found calling at night from shallow grassy puddles or flooded cultivated fields. Calling activity typically starts after 22:00, even though males may be at calling sites earlier. Males have been found calling in syntopy with *P. cooperi* and *P. levenorum*. Genetic analyses have shown that *P. levenorum* and *P. robeensis* sp. nov. hybridize where their ranges overlap.

#### Advertisement call.

The call of *Ptychadena robeensis* sp. nov. (5 males, 101 calls) is composed of a single pulsed note of 534 ± 53 ms in duration, containing 9.8 ± 1 regularly-spaced pulses. Pulse amplitude increases gradually until 292 ± 19 ms, after what it decreases. Call peak frequency is 2876 ± 74 Hz with a slight increase in frequency within the call.

The call of *P. robeensis* sp. nov. can be distinguished from the calls of all other members of the *P. neumanni* complex, except for *P. delphina* sp. nov., by its regularly spaced pulses and long inter-pulses intervals (57 ± 5 ms). A higher frequency allows discriminating the calls of *P. robeensis* sp. nov. and *P. delphina* sp. nov.

### Ptychadena beka sp. nov.

Taxon classificationAnimaliaAnuraPtychadenidae

BE21FD2A-9D41-5C90-9D56-E7446862297C

http://zoobank.org/CCF36B05-F7FC-436B-8858-765B6F682127

#### Type material.

***Holotype*.** Adult male (SB291) collected by S. Goutte and J. Reyes-Velasco on 5 June 2018 southwest of Nekemte (8.9950°N, 36.4955°E, 2213 m a.s.l.). ***Paratypes*.** one male (15–5) collected by X. Freilich, J. Reyes-Velasco and S. Boissinot on 3 August 2015 west of Holeta Genet (9.0508°N, 38.4312°E, 2433 m a.s.l.), one male (15–283) collected by X. Freilich, J. Reyes-Velasco and S. Boissinot on 14 August 2015 southwest of Nekemte (8.9742°N, 36.4906°E, 2243 m a.s.l.), one female (16–1) collected by J. Reyes-Velasco and S. Boissinot on 4 July 2016 west of Holeta Genet (9.05078°N, 38.4312°E, 2386 m a.s.l.), one female (SB268) collected by S. Goutte and J. Reyes-Velasco on 3 June 2018 northwest of Bonga (7.5085°N, 36.0637°E, 2038 m a.s.l.), 3 females (SB270, SB272 and SB276) collected by S. Goutte and J. Reyes-Velasco on 3 June 2018 south of Ambo (8.9671–8.9922°N, 37.7951–37.8471°E, 1945–2170 m a.s.l.), one female (SB282) collected by S. Goutte and J. Reyes-Velasco on 4 June 2018 southwest of Nekemte (8.9950°N, 36.4955°E, 2213 m a.s.l.), one female (SB287) and one male (SB292) collected by S. Goutte and J. Reyes-Velasco on 5 June 2018 southwest of Nekemte (8.9950°N, 36.4955°E, 2213 m a.s.l.), one male (SB387) collected by S. Goutte and J. Reyes-Velasco on 11 June 2018 south of Gech’a (7.5544°N, 35.4148°E, 1936 m a.s.l.), one male (SB471) collected by S. Goutte and Y. Bourgeois on 21 June 2018 east of Iteya (8.1275°N, 39.2732°E, 2138 m a.s.l.), one female (SB566) collected by S. Goutte and Y. Bourgeois north of Gohatsion (10.01967°N, 38.2494°E, 2448 m a.s.l.), one male (SB576) collected by S. Goutte and Y. Bourgeois on 7 July 2018 north of Gebre Guracha (9.8664°N, 38.3758°E, 2596 m a.s.l.), two males (SB614, SB617) collected by S. Goutte and Y. Bourgeois on 14 July 2018 east of Holeta Genet (9.0682°N, 38.5214°E, 2397 m a.s.l.). All specimens are deposited at ZNHM.

#### Diagnosis.

**Figure 18.**
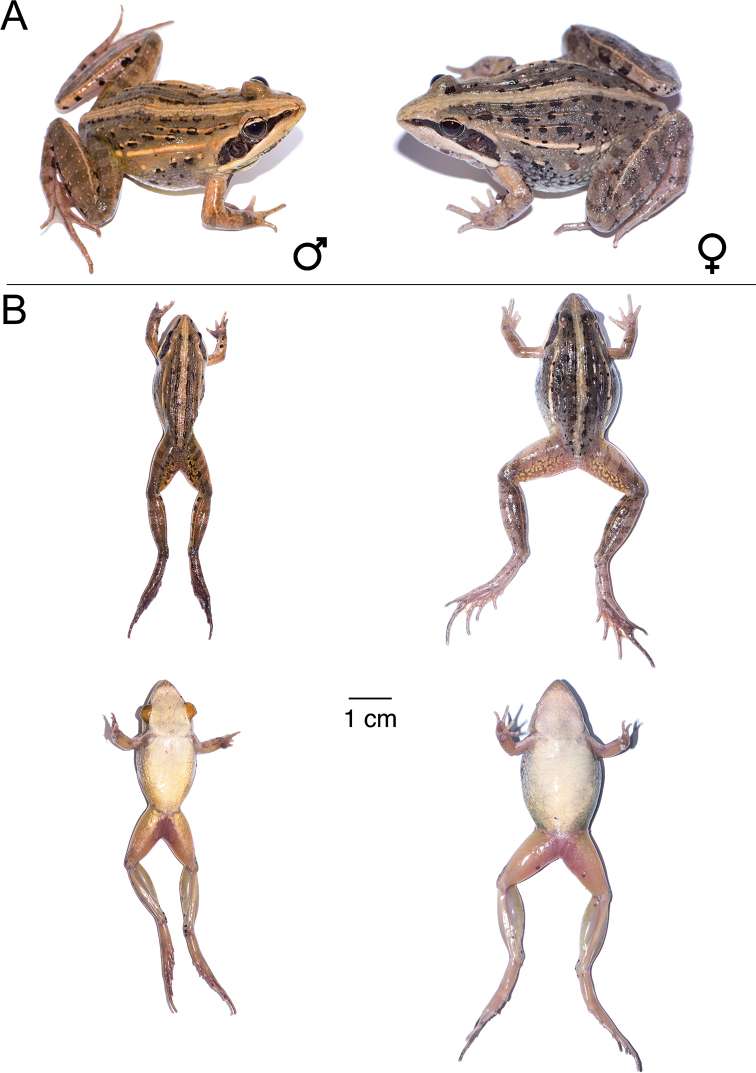
*Ptychadena beka* sp. nov. **A** live male holotype (SB291; left) and female paratype (SB270, right) **B** dorsal and ventral views of the same individuals (male left, female right) after euthanasia and before fixation.

A medium-sized species (male (9) SVL 37.9 ± 2.6 mm, female (8) SVL 43.2 ± 2.9 mm) of the *neumanni* species group (Fig. 18), distinguished by the following combination of characters: (1) relatively short hind limbs (male TL/SVL 0.51 ± 0.03, female TL/SVL 0.57 ± 0.03), (2) tympanum with a light vertical bar, (3) medium or wide vertebral stripe always present, (4) vocal sacs of most males are bicolored, from light to dark grey posteriorly and from yellow to cream anteriorly, rarely, they are light grey, (5) forearms of adult males not thickened.

#### Comparison.

*Ptychadena beka* sp. nov. is smaller than *P. cooperi* and *P. goweri* and larger than *P. nana* and *P. robeensis* sp. nov. Hand, feet, tibias, thighs and snout are of similar dimensions than *P. amharensis* and shorter than those of *P. delphina* sp. nov. and *P. goweri*. *Ptychadena beka* sp. nov. can be distinguished from *P. amharensis* by a wider inter-orbital distance. It can be distinguished from *P. erlangeri* by a larger tympanum, the light stripe or blotch on the tympanum and the bicolored vocal sacs in adult males. *Ptychadena beka* sp. nov. differs from *P. levenorum* by longer thighs, larger tympanum and longer snout. The head is wider and the tibias are longer than in *P. doro* sp. nov.

#### Description of the holotype.

A medium-sized (SVL 36.3 mm) adult male (Fig. 18). Head slightly wider than long. Snout projecting beyond the lower jaw. Interorbital distance 0.62 × the eye diameter. Internarial distance 1.12 × interorbital distance. Tympanum 0.67 × eye diameter. Finger tips not expanded but rounded, with very small subarticular tubercles. Finger formula: I<II<IV<III. Hand free of webbing, palmar tubercle absent. Nuptial pads light grey. Hindlimbs moderately elongated, with tibia length 0.51 × snout-vent length. Foot as long as thigh and shorter than tibia (FL/TL0.97). Toe tips rounded. Subarticular tubercles extremely small. Inner metatarsal tubercle present, external absent. Toe formula: I<II<V<III<IV. Foot webbing formula: Ie(1), IIi/e(1–1), IIIi/e(1–2), IVi/e(2–2), Vi(2). Two light brown, continuous lateral ridges, two continuous and four interrupted dorsal ridges. No vertebral nor sacral ridges. No warts on the body. Small and round light brown warts on tibias and thighs.

#### Coloration of the holotype in life.

Dorsal ground color brown with a few small, elongated dark brown blotches symmetrically distributed on the dorsolateral ridges. Wide light brown vertebral stripe, doubled with a thin, clearer line from the tip of the snout to the vent. Dark brown canthal stripe from the tip of the snout to the back of the jaw. Faint light vertical stripe on the otherwise brown tympanum. Upper and lower lip cream with irregular brown markings.

Iris bicolored, with upper third light silver-grey, and the lower two thirds dark brown. Small irregular dark olive brown blotches on greyish flanks. Throat and chest cream, ventrum, ventral sides of the thighs and of the tibias light yellow. Two small symmetrical dark brown blotches on the antero-ventral side of the shoulders. Thin, light longitudinal stripe on the tibias. Few white round warts on the tibias. Hind limbs brown with dark olive bars over the thighs, tibias, and feet. A few very small round white dots around the groin. Back of the thighs dark brown irregularly molted with yellow. Vocal sacs dark grey anteriorly, yellow posteriorly.

#### Coloration of the holotype in preservative.

Dorsal ground color grey with a few oval dark brown blotches symmetrically distributed on the dorsolateral ridges. Wide light brown vertebral stripe, doubled with a lighter thin line from the tip of the snout to the vent. Dark brown canthal stripe from the tip of the snout to the back of the jaw. Faint vertical stripe on the otherwise brown tympanum. Upper lip and lower lip cream heavily blotched with grey. Flanks brown with a few black blotches underlined with a cream bar. Throat, ventrum, ventral side of the thighs and tibias cream, with the chest and throat dusted with grey. Two symmetrical dark brown blotches on the ventral side of the shoulders. Faint, thin longitudinal stripe on the tibias. Faint brown bars on the thigh and tibia. A few small round white spots forming a line on the posterior side of tibia and foot. Back of thighs molted light and dark brown. Vocal sacs dark grey anteriorly, cream posteriorly. Nuptial pads light grey.

#### Variations.

In life, background color varies from yellowish brown to greyish brown. Dorsal ridges vary in number and definition. In all examined specimens, median ridges were continuous from the back of the head to the groin. Postpalpebral fold usually interrupted in the middle of the back, sometimes continuing on the lower back by a ridge or multiple aligned warts. Short latero-dorsal fold almost always present, sometimes fractioned. Lateral ridges, briefly interrupted in a few specimens, most often continuous, from orange-brown to cream. All specimens examined had small dark brown or black blotches distributed symmetrically on the dorsal ridges, in very few specimens, those blotches were very small or barely visible. All examined individuals have a wide, generally around a lighter thin stripe. The light blotch on the tympanum is generally small and more conspicuous in some specimens than in others. The thin cream longitudinal stripe on the tibia may be more or less conspicuous and extended to half of the thigh in some individuals. Tibias, thighs and feet posteriorly barred with more or less defined brown or light brown markings. Yellow and brown marbling on the posterior side of the thighs more or less contrasted and almost absent in some individuals. Ventrum and throat uniformly white to light yellow. Vocal sacs grey, bicolored cream to yellow and light grey to grey. Small warts over the back and flanks in ca. 10% of adult males examined.

#### Etymology.

The specific name corresponds to the translation of the word “enough” or “that’s it” in Amharic (በቃ), in reference to the controversial taxonomic history of the group that we hope has come to an end. It is an invariable noun used in apposition.

#### Habitat, distribution, and natural history.

*Ptychadena beka* sp. nov. has a wide distribution range extending both sides of the Great Rift Valley, although most records were made west of the GRV (6.14–10.01°N, 35.41–39.27°E). It is restricted to moderate altitudes, from 1695 m to 2596 m a.s.l. (based on 106 barcoded individuals). In the north, its range is limited by the Blue Nile valley and the northernmost specimens have been found just north of Gohatsion. The westernmost population has been found in Gech’a. Two populations have been found southeast of the GRV, near Iteya and south of Irba Muda. GPS coordinates for all examined specimens are given in Suppl. material 3: Table S1. *Ptychadena beka* sp. nov. is found in syntopy with *P. erlangeri* at the higher end of its altitudinal range, notably near Fitche, Holeta, between Ambo and Wonchi, and possibly near Assela. Within the genus, *P. beka* sp. nov. is also sympatric with *P. neumanni*, *P. delphina*, and *P. doro* in the west.

Males are found calling at in shallow roadside puddles or agricultural fields. Males can be found vocalizing very close from one another, sometimes in important numbers. Calling activity depends on rainfall and is highest during rainy months. Calling usually starts after 22:00, and sometimes as late as 02:00 in dry weather, and ceases before dawn. Numerous, small bicolored eggs are laid in the same water body.

#### Advertisement call.

The call of *Ptychadena beka* sp. nov. (5 males, 128 calls) is composed of a single pulsed note of 447 ± 112 ms in duration, containing 31 ± 10.6 pulses. Within calls, pulses are grouped by 3–5 pulses, with the first and the last pulses of each pulse group notably lower in amplitude than the other pulses. Low-amplitude single pulses are sometimes present between pulse groups. Amplitude increases gradually during the call, peaking at 266 ± 78 ms, after which it drops. Call dominant frequency is 2491 ± 129 Hz.

The call of *P. beka* sp. nov. can be distinguished from those of *P. cooperi*, *P. amharensis*, *P. doro* sp. nov., and *P. neumanni* (type A and B) by the distinguishable pulses composing the calls. It is also distinct from the calls of *P. delphina* sp. nov. and *P. robeensis* sp. nov. by its short inter-pulse intervals. The call of *P. beka* sp. nov. differs from the calls of *P. delphina* sp. nov., *P. erlangeri*, *P. levenorum*, and *P. goweri* by its higher dominant frequency. Finally, the call of *P. beka* sp. nov. differs from the call of *P. nana* and *P. erlangeri* by its longer duration.

### Ptychadena delphina sp. nov.

Taxon classificationAnimaliaAnuraPtychadenidae

C980B0B6-3C5C-503A-A82F-C846A6ECF097

http://zoobank.org/8BFAD046-6E3B-4622-B488-D4483FA2BE31

#### Type material.

***Holotype*.** Adult male (SB310) collected on 7 June 2018 by S. Goutte and J. Reyes-Velasco between Dembi and Gechi, Oromia, Ethiopia (8.2195°N, 36.446°E, 2064 m a.s.l.). ***Paratypes*.** One female (16–242) and one male (16–241) collected 22 July 2016 by X. Freilich, J. Reyes-Velasco and S. Boissinot west of Bedele (8.3746°N, 36.2596°E, 1876 m a.s.l.), one female (SB295) collected on 6 June 2018 by S. Goutte and J. Reyes-Velasco west of Bedele (8.4330°N, 36.3176°E, 1942 m a.s.l.), two males (SB313 and SB314) collected on 7 June 2018 by S. Goutte and J. Reyes-Velasco between Dembi and Gechi (8.2195°N, 36.4460°E, 2064 m a.s.l.), one female (SB341) collected on 9 June 2018 by S. Goutte and J. Reyes-Velasco west of Gore (8.1769°N, 35.3627°E, 1612 m a.s.l.), 3 females(SB355, SB356 and SB357) and two males (SB354 and SB363) collected on 10 June 2018 by S. Goutte and J. Reyes-Velasco south of Gore (8.0802°N, 35.5239°E, 1903 m a.s.l.). All specimens are deposited at ZNHM.

#### Diagnosis.

**Figure 19.**
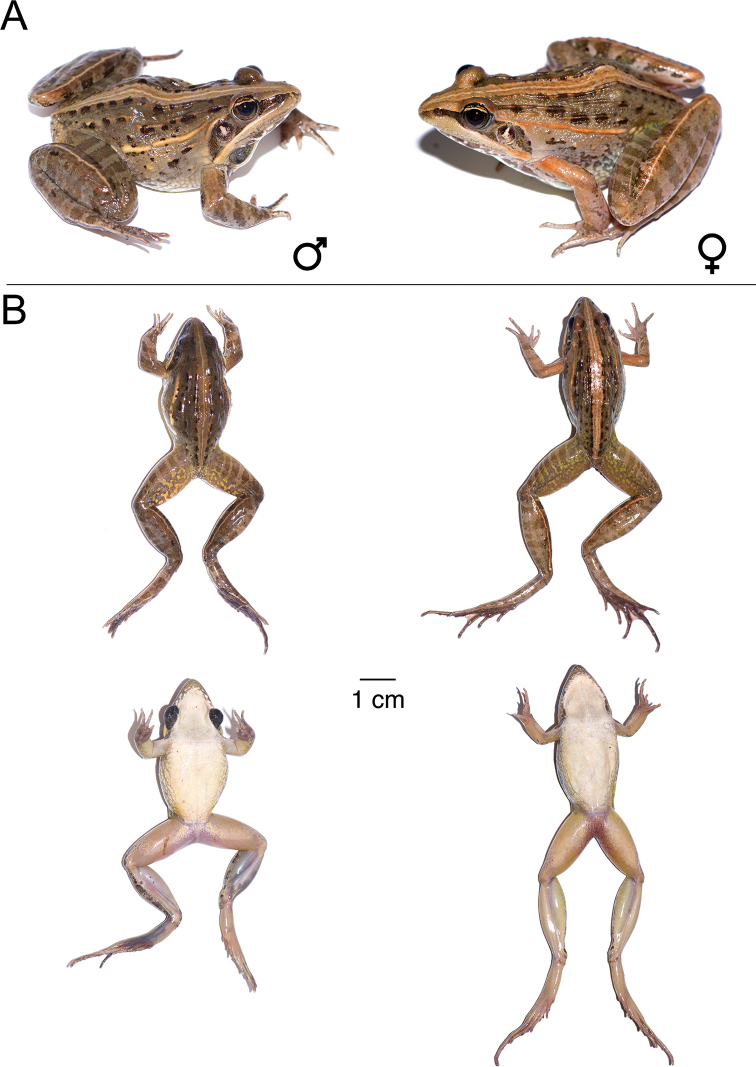
*Ptychadena delphina* sp. nov. **A** live male holotype (SB310; left) and female paratype (SB341, right) **B** dorsal and ventral views of the same individuals (male left, female right) after euthanasia and before fixation.

Large member (male (6) SVL 40.1 ± 2.9 mm, female (6) SVL 47.2 ± 2.3 mm) of the *neumanni* species group (Fig. 19) distinguished by the following combination of characters: (1) moderately long hind limbs (male TL/SVL 0.53 ± 0.02, female TL/SVL 0.55 ± 0.03), (2) long forearms (FLL/SVL 0.20 ± 0.01), (3) eye close to one another (male IOD/HW 0.17 ± 0.02, female IOD/HW 0.20 ± 0.03), (4) light vertical stripe on the tympanum, (5) vocal sacs are dark grey or dark grey posteriorly and lighter anteriorly.

#### Comparison.

*Ptychadena delphina* sp. nov. is smaller than *P. cooperi* and larger than *P. nana*, *P. erlangeri*, *P. levenorum*, and *P. robeensis* sp. nov. This species has shorter hind limbs and feet than *P. goweri* but longer than *P. beka* sp. nov. and *P. amharensis* (see Suppl. material 3: Table S2). The length of its forearms is also greater than in *P. beka* sp. nov. and *P. amharensis*. *Ptychadena delphina* sp. nov. can be distinguished from *P. goweri* by shorter hands, shorter snout, and pigmented vocal sacs in adult males. Eyes are closer to one-another than *P. goweri*, but further apart than in *P. amharensis*. Tympanum larger than *P. doro* sp. nov.

#### Description of the holotype.

Relatively large (SVL 44.2 mm) male (Fig. 19). Head wider than long (HL/HW 0.87). Snout projecting beyond the lower jaw. Interorbital distance 0.54 × eye diameter. Internarial distance 1.68 × interorbital distance. Tympanum 0.71 × eye diameter. Finger tips not expanded but rounded, with small subarticular tubercles.

Finger formula: I<II<IV<III. Hand free of webbing. Hindlimbs elongated, with tibia length 0.55 × snout-vent length. Foot longer than thigh (FL/THL 1.27) and slightly longer than tibia (FL/TL 1.02). Toe tips rounded. Subarticular tubercles small and round. Inner metatarsal tubercle present, external absent. Fourth toe on the left foot amputated. Toe formula: I<II<V<III<IV. Foot webbing formula: Ie(minimal), IIi/e(minimal–1), IIIi/e(2–2), IVi/e(2–2), Vi(2). Two light lateral ridges, continuous on the right side and discontinuous on the left side. Two continuous dorsal ridges and two interrupted dorsolateral ridges. No vertebral nor sacral ridges. Body and eyelids covered with minute transparent warts. Nuptial pad developed along finger I. Tongue longer than wide, free for half of its length, bifurcated at the end. Vomerine, maxillary and premaxillary teeth present.

#### Coloration of the holotype in life.

Dorsal ground color brown with elongated dark brown blotches symmetrically distributed on the dorsal ridges. Wide light grey-brown vertebral stripe, doubled with a thin, clearer stripe from the tip of the snout to the vent. Dark brown canthal stripe from the tip of the snout to the back of the jaw. Light vertical blotch on the otherwise brown tympanum. Upper and lower lip cream to light brown with irregular brown markings. Iris dark brown on the lower two thirds, light golden brown above, separated by a cream horizontal stripe.

A few irregular dark brown blotches on the light grey flanks. Throat and ventrum cream with a few small light grey dots under the chin. Two small symmetrical dark brown blotches on the antero-ventral side of the shoulders. Thin light longitudinal stripe on the tibias and lower half of the thighs. Hind limbs brown with dark bars over the thighs, tibias, and feet. A few small round white dots around the groin. Back of the thighs dark brown irregularly molted with yellow. Vocal sacs uniformly dark grey.

#### Coloration of the holotype in preservative.

Dorsal ground color dark brown with a few small, oval dark brown blotches symmetrically distributed on the dorsolateral ridges. Wide lighter brown vertebral stripe, doubled with a thin, clearer line from the tip of the snout to the vent. Dark brown canthal stripe from the tip of the snout to the back of the jaw. Light vertical stripe on the otherwise brown tympanum. Upper lip grey on the anterior half and cream on posterior half. Lower lip grey with irregular cream spots. Flanks brown with a few irregular small dark brown blotches. Throat, ventrum, ventral side of the thighs and tibias uniformly cream with the throat and chest lightly dusted with grey. Two symmetrical dark brown blotches on the ventral side of the shoulders. Thin, light longitudinal stripe on the tibias. Faint brown bars on the thighs. Back of thighs molted light grey and brown. Vocal sacs dark grey. Nuptial pads cream.

#### Variations.

In life, background color varies from light to dark brown. Dorsal ridges vary in number and definition. Median ridges may be continuous from eye level to the groin, or interrupted and be present only along half the back. Postpalpebral fold interrupted in the middle of the back, sometimes continuing on the lower back. Short laterodorsal fold almost always present. Lateral ridges generally non-interrupted until the lower third of the back and from orange to cream. All specimens examined had small dark brown or black blotches distributed symmetrically on the dorsal ridges. Inguinal area more or less conspicuously yellow. Vertebral stripe may be thin or wide, generally around a lighter thin stripe. Tympanum blotch may be more or less conspicuous depending on the individual.

The thin cream longitudinal stripe on the tibia may be extended to the thigh or half of the thigh and the foot in some individuals. Tibias, thighs, and feet posteriorly barred with more or less defined brown or light brown markings. Thighs posteriorly marbled with light to dark brown and yellow. Ventrum and throat uniformly white to light yellow. Vocal sacs grey to dark grey or bicolored cream and grey. Small warts over the back and flanks in ca. 20% of adult males.

#### Etymology.

The specific name originates from the Latin *delphinus*, dolphin, in reference to the advertisement call of the species resembling a dolphin’s clicking sound. We have Latinized *delphinus* into the adjective *delphina* to be in accordance with the gender of genus *Ptychadena*. The advertisement call best distinguishes *P. delphina* sp. nov. from *P. doro* sp. nov.

#### Habitat, distribution, and natural history.

The distribution range of *Ptychadena delphina* sp. nov. is mostly restricted to mid-elevation forests (1612 to 2064 m a.s.l.), west of the GRV and north of the Geba River (tributary of the Baro River). However, two individuals (XF13–283 and XF13–285) collected in 2013 by X. Freilich and S. Boissinot in Asgori, between Addis Ababa and Ambo (8.9799°N, 38.0241°E, 2370 m a.s.l.) clustered with *Ptychadena delphina* in phylogenetic analyses based on four molecular markers (Freilich et al. 2016). If the molecular results are not caused by introgression between *P. delphina* sp. nov. and *P. beka* sp. nov, which occurs in the area, and if these two individuals represent a real population, this is the easternmost and highest known population of *P. delphina* sp. nov. The habitat at this locality is also quite different from the rest of *P. delphina* sp. nov. distribution range, as it is composed of open agricultural fields and not forest. No other individual of the species was collected in the multiple sampling campaigns subsequently conducted, and this population remains to be confirmed. Beside these two individuals, the easternmost populations have been found south of Bedele, while, in the west, Individuals have been collected by Uka, west of Gore. The southernmost individuals were found in Bichano, just north of the Geba River.

Males of *P. delphina* sp. nov. call at night in flooded grassland ponds or puddles, or in rainwater-filled holes on the road. Within the genus, *P. delphina* sp. nov. is found in sympatry with *P. beka* sp. nov., *P. doro* sp. nov., and *P. neumanni*. Males of *P. delphina* sp. nov. have been found calling jointly with *P. doro* sp. nov.

#### Advertisement call.

To the human ear, the call of *Ptychadena delphina* sp. nov. (4 males, 33 calls) resembles a dolphin’s series of clicks. It is composed of a single note of 504 ± 92 ms in duration, containing 8.4 ± 1.4 pulses, which are clearly distinct and at regular intervals. Amplitude increases regularly within the note until 384 ± 129 ms, where it decreases. As in other Ethiopian *Ptychadena* species, call repetition rate is highly variable and dependent of the social context. Call dominant frequency is 2327 ± 147 Hz, with a gradual increase in dominant frequency within the call.

The call of *Ptychadena delphina* sp. nov. is easily distinguishable from the calls of all other *Ptychadena* from the Ethiopian highland by its well defined and regularly spaced pulses, except for the call of *P. robeensis*, which presents a similar structure. The call *P. robeensis* can however be distinguished by its higher dominant frequency (2876 ± 74 Hz), related to the species’ smaller body size. It is worth noting that the call of the closely related syntopic species *P. doro* sp. nov. is remarkably different both in temporal and spectral features, while the two species are morphologically extremely similar. The two species were thus named after their respective calls, which constitute their most distinguishable traits.

### Ptychadena doro sp. nov.

Taxon classificationAnimaliaAnuraPtychadenidae

0705404D-7B40-56A1-8856-5655B54204C3

http://zoobank.org/CA0740EE-5B73-479B-8202-E61F1C29788A

#### Type material.

***Holotype*.** An adult male (SB328) collected on 8 June 2018 by S. Goutte and J. Reyes-Velasco between Dembi and Gechi, Oromia, Ethiopia (8.2195°N, 36.446°E, 2064 m a.s.l.). ***Paratypes*.** 14 males: one male (15–260) collected on 15 August 2015 by X. Freilich, J. Reyes-Velasco and S. Boissinot south of Gech’a (7.4213°N, 35.3993°E, 2316 m a.s.l.), two males (16–198, 16–200) collected on 17 July 2016 by J. Reyes-Velasco and S. Boissinot northwest of Jimma (7.7307°N, 36.6926°E, 2218 m a.s.l.), two females (16–344, 16–350) and 3 males (16–352, 16–353, 16–361) collected on 21 July 2016 by J. Reyes-Velasco and S. Boissinot south of Gech’a (7.4212°N–7.4393°N, 35.3992–35.4047°E, 2240–2304 m a.s.l.), one male (SB247) collected on 24 April 2018 by S. Goutte and J. Reyes-Velasco west of Jimma (7.5449°N, 36.582°E, 2272 m a.s.l.), two females (SB298, SB309) and 3 males (SB299, SB311, SB312) collected on 7 June 2018 by S. Goutte and J. Reyes-Velasco between Dembi and Gechi (8.2195–8.2524°N, 36.446–36.4465°E, 2064–2198 m a.s.l.), one female (SB327) collected on 8 June 2018 by S. Goutte and J. Reyes-Velasco between Dembi and Gechi (8.2195°N, 36.446°E, 2064 m a.s.l.), one female (SB346) collected on 9 June 2018 by S. Goutte and J. Reyes-Velasco west of Gore (8.1645°N, 35.3819°E, 1654 m a.s.l.), one male (SB386) collected on 11 June 2018 by S. Goutte and J. Reyes-Velasco south of Gech’a (7.5544°N, 35.4148°E, 1936 m a.s.l.), one male (SB408) collected on 13 June 2018 by S. Goutte and J. Reyes-Velasco south of Gech’a (7.5532°N, 35.4158°E, 1940 m a.s.l.). All specimens are deposited at ZNHM.

#### Diagnosis.

**Figure 20.**
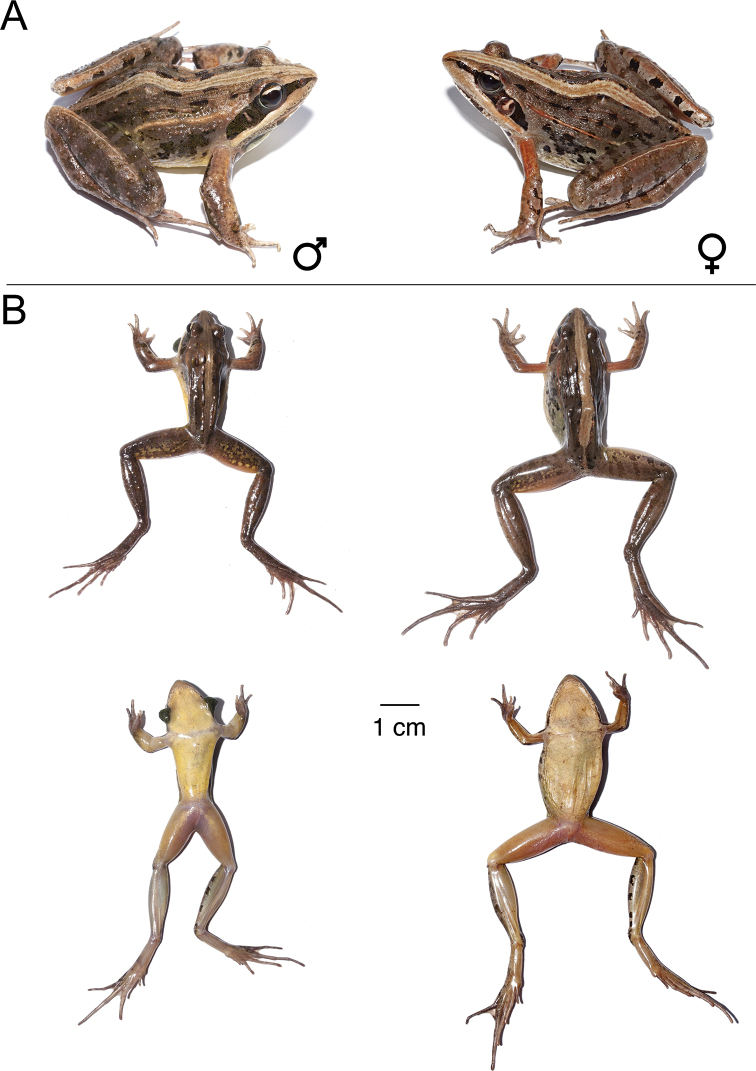
*Ptychadena doro* sp. nov. **A** live male holotype (SB328; left) and female paratype (SB327, right) **B** dorsal and ventral views of the same individuals (male left, female right) after euthanasia and before fixation.

Moderately large (male (13) SVL 35.8 ± 2.2 mm, female (6) SVL 42.4 ± 5.7 mm) member of the *neumanni* species group (Fig. 20) distinguished by the following combination of characters: (1) long hind limbs (male TL/SVL 0.58 ± 0.03, female TL/SVL 0.64 ± 0.11), (2) long hands (male HAL/SVL 0.24 ± 0.02, female HAL/SVL 0.25 ± 0.05), (3) long snout (male SL/SVL 0.16 ± 0.01, female SL/SVL 0.16 ± 0.02), (4) relatively small tympanum (male TD/ED 0.64 ± 0.09, female TD/ED 0.63 ± 0.1), (5) light vertical stripe on the tympanum, (6) vocal sacs are grey or dark grey posteriorly and lighter anteriorly,

#### Comparison.

*Ptychadena doro* sp. nov. can be distinguished from all medium sized Ethiopian *Ptychadena* by its long feet and smaller tympanum. It can be further distinguished from *P. beka* sp. nov. by a narrower head and longer tibias. Snout is longer, inter-nares distance is greater and hands are longer than in *P. erlangeri* and *P. levenorum*. It can be distinguished from *P. amharensis* and *P. levenorum* by its larger inter-orbital distance. *Ptychadena doro* sp. nov. can be distinguished from *P. delphina* sp. nov. by a smaller body, a narrower head and shorter inter-orbital distance.

#### Description of the holotype.

A medium-sized (SVL 36.4 mm) adult male with long hind limbs (TL/SVL 0.58; Fig. 20, Suppl. material 3: Table S1). Head as long as wide. Snout projecting beyond the lower jaw. Interorbital distance 0.60 × the eye diameter. Internarial distance 1.08 × interorbital distance. Tympanum 0.63 × eye diameter. Finger tips not expanded but rounded, with very small subarticular tubercles. Finger formula: I<II<IV<III. Hand free of webbing, palmar tubercle absent. Nuptial pads light grey to cream, extending dorsally to the second finger. Hindlimbs elongated, with tibia length 0.57 × snout-vent length. Foot longer than thigh and slightly longer than tibia (FL/THL 1.20, FL/TL 1.01). Toe tips rounded. Subarticular tubercles extremely small. Inner metatarsal tubercle present, external absent. Toe formula: I<II<V<III<IV. Foot webbing formula: Ie(1), IIi/e(1–1), IIIi/e(1–2), IVi/e(2–2), Vi(2). Two light brown, continuous lateral ridges, two continuous and four interrupted dorsal ridges. No vertebral nor sacral ridges. Very small, translucent warts on the body and small round warts on tibias and feet. Tongue free for half of its length, bifurcated at the end. Vomerine, maxillary and premaxillary teeth present.

#### Coloration of the holotype in life.

Dorsal ground color brown with a few elongated dark brown blotches symmetrically distributed on the dorsolateral ridges. Wide light grey-brown vertebral stripe, doubled with a thin, clearer stripe from the tip of the snout to the vent. Dark olive brown canthal stripe from the tip of the snout to the back of the jaw. Light vertical stripe on the otherwise brown tympanum. Upper and lower lip cream to light brown with irregular brown markings.

Iris bicolored, with upper third light silver-grey, and the lower two thirds dark brown. Irregular dark olive brown blotches fused into a large undefined mark on the flanks. Throat, ventrum, ventral side of the thighs and tibias uniformly light yellow. Two small symmetrical dark brown blotches on the antero-ventral side of the shoulders. Thin, barely visible, light longitudinal stripe on the tibias. Few white round warts on the tibias. Hind limbs brown with dark olive bars over the thighs, tibias, and feet. A few very small round white dots around the groin. Back of the thighs dark brown irregularly molted with yellow. Vocal sacs uniformly dark grey.

#### Coloration of the holotype in preservative.

Dorsal ground color grey with a few oval dark brown blotches symmetrically distributed on the dorsolateral ridges. Wide lighter brown vertebral stripe from the tip of the snout to the vent. Dark brown canthal stripe from the tip of the snout to the back of the jaw. Faint vertical stripe on the otherwise brown tympanum. Upper lip and lower lips cream heavily blotched with grey. Flanks brown with a few irregular small dark brown blotches. Throat, ventrum, ventral side of the thighs and tibias uniformly cream. Two symmetrical grey blotches on the ventral side of the shoulders. Faint, thin longitudinal stripe on the tibias. No distinct bars on the thighs or tibias. A few small round white spots forming a line on the posterior side of tibias. Back of thighs molted yellowish cream and brown. Vocal sacs dark grey. Nuptial pads light grey.

#### Variations.

In life, background color varies from yellowish brown to dark brown. Dorsal ridges vary in number and definition. In all examined specimens, median ridges were continuous from eye level to the groin. Postpalpebral fold usually interrupted in the middle of the back, sometimes continuing on the lower back by a ridge or multiple aligned warts. Short laterodorsal fold almost always present, sometimes fractioned. Lateral ridges generally briefly interrupted once or twice and from orange to cream. All specimens examined had small dark brown or black blotches distributed symmetrically on the dorsal ridges. Inguinal area yellowish in some individuals. Most individuals have a wide, generally around a lighter thin stripe, some individuals have a thin vertebral stripe only. All examined specimens had a very conspicuous cream blotch on the tympanum.

The thin cream longitudinal stripe on the tibia may be more or less conspicuous and extended to the thigh or half of the thigh and the foot in some individuals. Tibias, thighs, and feet posteriorly barred with more or less defined brown or light brown markings. Yellow and brown marbling on the posterior side of the thighs almost absent in some individuals. Ventrum and throat uniformly light yellow, sometimes very lightly dusted with light grey on the throat. Vocal sacs grey, bicolored cream and grey in some individuals, and rarely light grey. Small warts over the back and flanks in ca. 50% of adult males.

#### Etymology.

The species name is the Amharic translation of chicken (ዶሮ), in reference to the advertisement call of the species resembling a chicken’s song. The advertisement call distinguishes best *Ptychadena doro* sp. nov. from the syntopic species *P. delphina* sp. nov.

#### Habitat, distribution, and natural history.

The distribution range of *Ptychadena doro* sp. nov. is restricted to mid-elevations (1654 to 2318 m a.s.l.), west of the GRV. The easternmost populations have been found around Jimma and south of Bedele. Individuals have been collected west of Metu and between Metu and Tippi. Males of *P. doro* sp. nov. call at night in flooded grassland ponds or puddles, or in rainwater-filled holes on the road. Within the genus *Ptychadena*, *P. doro* sp. nov. is found in sympatry with *P. beka* sp. nov., *P. delphina* sp. nov. and *P. neumanni*. Males of *P. doro* sp. nov. have been found calling jointly with males of *P. delphina* sp. nov.

#### Advertisement call.

The call of *Ptychadena doro* sp. nov. (3 males, 21 calls) is reminiscent of a chicken call. It is composed of a single, pulsed note of 411 ± 41 ms in duration. Pulses are partly fused without any silent intervals between them. Amplitude increases during most of the note (peak amplitude at 295 ± 39 ms) and decreases abruptly at the end of the note. As in other Ethiopian *Ptychadena* species, call repetition rate is highly variable and dependent of the social context. Call dominant frequency is 1966 ± 105 Hz, with a gradual increase in frequency within the note. Frequency bandwidth is remarkably narrower than that of the calls of the other species of the *P. neumanni* complex (415 ± 31 Hz), resulting in a more tonal sound. The call of *P. doro* sp. nov. is easily distinguishable from the call of all other species of the *P. neumanni* complex by its single note, tonal call composed of indistinct pulses.

### Ptychadena goweri

Taxon classificationAnimaliaAnuraPtychadenidae

Smith, Noonan & Colston, 2017

D81C7126-345D-57D4-A71B-845C742CC2E5

#### Type material.

***Holotype*.** An adult male (TJC224) collected on 10 December 2012 by T. J. Colston in Katcha, Bale National Park, Ethiopia (6.71779°N, 39.72572°E, 2375 m a.s.l.). ***Paratypes*.** Three juveniles (XF781, XF782, XF783) collected 7 August 2011 by X. Freilich and S. Boissinot north of Hagere Mariam, Oromia, Ethiopia (5.8027°N, 38.2705°E, 2323 m a.s.l.). All type specimens and examined material are deposited at ZNHM.

#### Material examined.

In addition to the holotype, we examined one male (TJC218) collected by T. J. Colston , one female (15–85) and two males (15–103, 15–105) collected on 7 August 2015 by X. Freilich, J. Reyes-Velasco and S. Boissinot in the Harenna forest (6.5866°N, 39.7417°E, 1778 m a.s.l.), one male (15–121) collected on 7 August 2015 by X. Freilich, J. Reyes-Velasco and S. Boissinot in the Harenna forest (6.6634°N, 39.7302°E, 2002 m a.s.l.), one female (15–425) and two males (15–426, 15–427) collected on 27 September 2015 by X. Freilich, J. Reyes-Velasco and S. Boissinot northwest of Kibre Mengist (5.9055–6.04546°N, 38.837–38.9334°E, 1745–2238 m a.s.l.), two males (15–448, 15–449) collected on 28 September 2015 by X. Freilich, J. Reyes-Velasco and S. Boissinot in Harenna forest (6.71925 N, 39.7202 E), two males (SB99 and SB100) collected on 10 April 2018 by S. Goutte and J. Reyes-Velasco in the Harenna forest (6.6634°N, 39.7302°E, 2440 m a.s.l.), two females (SB158, SB159) and two males (SB160, SB161) collected on 17 April 2018 by S. Goutte and J. Reyes-Velasco northwest of Kibre Mengist (6.0093°N, 38.8576°E, 2105 m a.s.l.), one female (SB807) collected on 7 May 2019 by S. Goutte in the Harenna forest (6.6640°N, 39.7301°E, 1992 m a.s.l.), one female (SB808) collected on 10 May 2019 by S. Goutte in the Harenna forest (6.7164°N, 39.7257°E, 2375 m a.s.l.).

#### Diagnosis.

**Figure 21.**
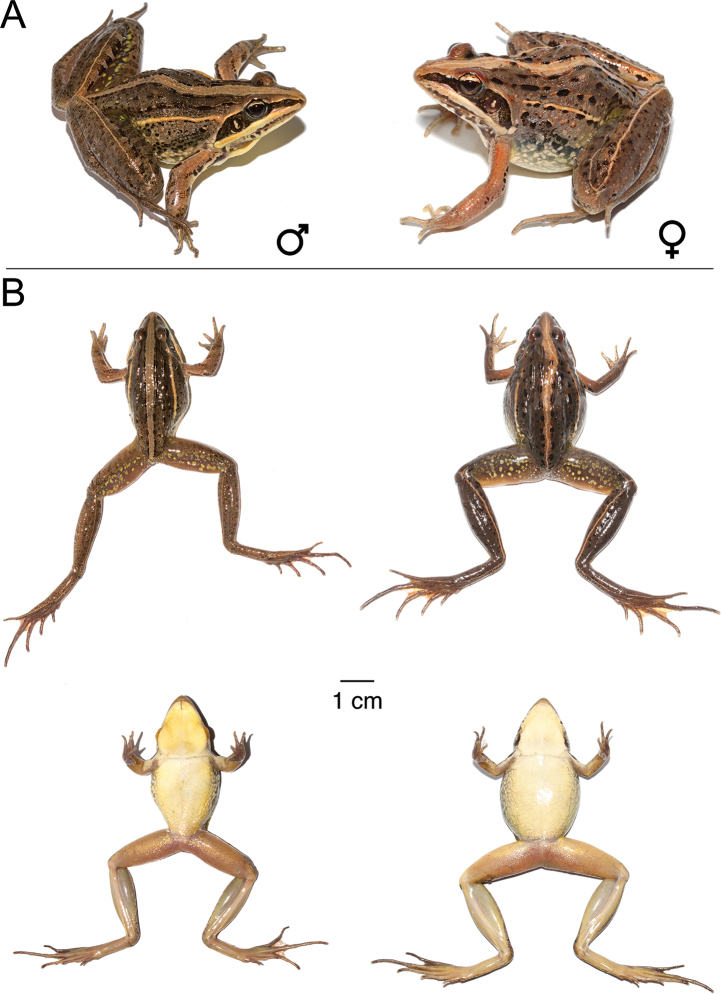
*Ptychadena goweri***A** Live male (SB99; left) and female (SB5807, right) **B** dorsal and ventral views of the same individuals (male left, female right) after euthanasia and before fixation.

Large species (male (15) SVL 42.4 ± 2.7 mm, female (6) SVL 51.2 ± 4.2 mm) from the *neumanni* species group (Fig. 21) distinguished by the following combination of characters: (1) long hind limbs (male TL/SVL 0.60 ± 0.05, female TL/SVL 0.59 ± 0.02) , (2) long feet (male FL/SVL 0.61 ± 0.05, female FL/SVL 0.58 ± 0.02), (3) long hands (male HAL/SVL 0.24 ± 0.03, female HAL/SVL 0.23 ± 0.01), (4) long head (male HL/SVL 0.38 ± 0.04, female HL/SVL 0.36 ± 0.04), (5) long snout (male SL/SVL 0.16 ± 0.02, female SL/SVL 0.15 ± 0.01), (6) vocal sacs light grey, cream or yellow, sometimes mottled with light grey, (7) male skin smooth.

#### Comparison.

Except for *P. cooperi*, the largest species of the *Ptychadena neumanni* complex. Body size alone distinguishes *Ptychadena goweri* from *P. nana*, *P. robeensis* sp. nov., *P. levenorum*, *P. erlangeri*, and *P. doro* sp. nov. Compared to the larger *Ptychadena* species of Ethiopian highlands, it has longer thigh, tibia, feet, hands, head, snout, and inter-orbital distance than *P. delphina* sp. nov., *P. beka* sp. nov., and *P. amharensis*. The light-colored vocal sacs of adult males distinguish *P. goweri* from *P. beka* sp. nov., *P. delphina* sp. nov., and *P. neumanni* and *P. cooperi*. The almost complete dorsal ridges distinguish further *P. goweri* from *P. cooperi*, which presents rows of short glandular folds on the dorsum.

#### Description of the holotype.

**Figure 22.**
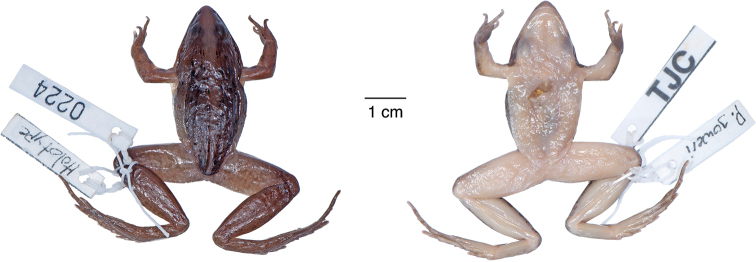
Holotype of *Ptychadena goweri*. Dorsal and ventral views of the male holotype (TJC224).

Relatively large (SVL 41.9 mm) male (Fig. 22) with long hind limbs (TL/SVL 0.58, Suppl. material 3: Table S3). Head as long as wide. Snout projecting beyond the lower jaw. Interorbital distance almost equal to the eye diameter. Internarial distance 0.88 × interorbital distance. Tympanum 0.85 × eye diameter. Finger tips not expanded but rounded, with moderate subarticular tubercles. Finger formula: I<II<IV<III. Hand free of webbing. Hindlimbs elongated, with tibia length 0.58 × snout-vent length. Foot slightly longer than thigh and tibia (FL/THL 1.14, FL/TL 1.05). Toe tips rounded. Subarticular tubercles small and round. Inner metatarsal tubercle present, external absent. Toe formula: I<II<V<III<IV. Foot webbing formula: Ie(1), IIi/e(1–1.5), IIIi/e(1.5–2), IVi/e(2–2), Vi(2). Two continuous lateral ridges, brown anteriorly and light brown posteriorly, barely visible, six interrupted dorsal ridges. No vertebral, nor sacral ridges. No wart on body or limbs. Tongue free for less than a third of its length, divided in two lobes. Vomerine, maxillary and premaxillary teeth present.

#### Coloration of the holotype in preservative.

Dorsal ground color brown with a few small, irregular oval dark brown blotches symmetrically distributed on the dorsolateral ridges. Wide light brown vertebral stripe, doubled with a thin, clearer stripe from the tip of the snout to the vent. Dark brown canthal stripe from the tip of the snout to the back of the jaw. Light vertical stripe on the otherwise brown tympanum. Upper and lower lip brown with irregular dark brown molting. Flanks grey anteriorly to light brown posteriorly with irregular dark brown blotches on the anterior half. Throat, ventrum, ventral side of the thighs and tibias uniformly cream with very light grey dusting on the throat. Two symmetrical dark brown blotches on the ventral side of the shoulders. Thin, barely visible, light longitudinal stripe on the tibias. Irregular and undefined brown markings on the thighs, tibias, and feet. Back of thighs molted light grey and brown. Vocal sacs mostly white, with slight grey dusting posteriorly.

#### Variations.

In life, background coloration varies from light brown, olive grey to dark brown. The vertebral stripe may be thin or wide doubled with a thin lighter stripe within it, and from cream to grey and light yellowish brown. The dark brown to black blotches on the dorsum vary in size and number but are always organized along the dorsal ridges. Some individuals present small, irregular black markings in between ridges. Dark markings on the flanks are quite variable between individuals, from a few large dark blotches to a multitude of smaller ones, covering mostly the antero-dorsal part of the flank. Dorsolateral ridges can be discontinued once or twice and can be cream, yellow, or brown-orange. Some individuals present a reddish-brown marking on top of the eyelid.

Iris bicolored, with upper third silver to golden and lower two thirds dark golden to dark brown. Cream or golden vertical blotch on the dark brown tympanum always present. The thin stripe on the tibia may extend on the thigh in some individuals. Forearms, thighs, tibias and feet are more or less clearly marked with dark brown bars. Vocal sacs always light in color (yellow, cream, or light grey), more or less dusted with grey on their dorsoposterior sides. Dorsal ridges may be more or less discontinuous.

#### Habitat, distribution, and natural history.

The distribution range of *Ptychadena goweri* is restricted to the southeast of the GRV, at elevation ranging from 1745 m to 2550 m a.s.l. The species is found in clearings in the Harenna forest, south of the Sanetti plateau, from Kibre Mengist to Irba Muda, and north of Hagere Mariam (5.8027–6.7193°N, 28.2705°E). Males are found calling at night in shallow puddles in clearings or grassy meadows. Within the genus, *Ptychadena goweri* is found in sympatry with *P. harenna*, *P. levenorum*, *P. neumanni*, and *P. erlangeri*.

#### Advertisement call.

The call of *Ptychadena goweri* (4 males, 32 calls) is composed of a single pulsed note of 634 ± 74 ms in duration, containing 33.5 ± 2.9 pulses. Pulses are grouped by 3.3 ± 1.5 pulses within each note and increase in amplitude up to 404 ± 65 ms, after what the amplitude decreases. Within pulses groups, the first pulse has the lowest, while the second pulse has generally the greatest amplitude. Call repetition rate is highly variable and dependent on the social context. The individuals we recorded produced calls in “bursts”, where several males were forming a short chorus, spaced by long silent intervals. Call dominant frequency is 2318 ± 86 Hz with a slight increase in frequency within notes.

Within the *P. neumanni* complex, the call of *P. goweri* can be distinguished from those of *P. cooperi*, *P. amharensis*, *P. doro* sp. nov., and *P. neumanni* (type A and B) by the distinguishable pulses composing the calls. Grouped pulses and short inter-pulses intervals (9 ± 2 ms within pulse groups) distinguish the call of *P. goweri* from those of *P. robeensis* sp. nov. and *P. delphina* sp. nov. The call of *P. goweri* can be further distinguished from those of *P. nana* and *P. erlangeri* by a longer duration.

### Ptychadena neumanni

Taxon classificationAnimaliaAnuraPtychadenidae

(Ahl, 1924)

F9418628-0C51-55C0-B4CA-4F978B36CC5E

Rana neumanni Ahl, 1924: 4.
Ptychadena neumanni – Perret 1980: 157.
Rana (Ptychadena) neumanni – Dubois 1981: 233.
Ptychadena (Ptychadena) neumanni – Dubois 1992: 316.

#### Lectotype by present designation.

One adult male (ZMB26879–1) collected on 2 February 1901 by Oscar Neumann in Gadat (Gofa), south Ethiopia. [Coordinates estimated by Largen (2001): 6.33°N, 36.83°E, 2000 m a.s.l., but see remarks below]. ***Paralectotypes*.** Two adult males (ZMB–57183 = ZMB26879–2 and ZMB–57184 = ZMB26879–3) collected by Oscar Neumann on the same date and location as the lectotype (ZMB26879–1).

#### Material examined.

Except for the type series, all examined specimens are deposited at ZNHM. In addition to the type series, we examined one male (15–173) collected by X. Freilich, J. Reyes-Velasco and S. Boissinot on 9 August 2015 in Wondo Genet (7.0833°N, 38.6381°E, 1896 m a.s.l.), one female (15–181) and four males (15–183, 15–191, 15–208, 15–209) collected by X. Freilich, J. Reyes-Velasco and S. Boissinot on 12 August 2015 northwest of Bonga (7.3076°N, 36.1226°E, 1861 m a.s.l.), one female (16–203) collected on 18 July 2016 by J. Reyes-Velasco and S. Boissinot southwest of Bonga (7.2542°N, 36.2628°E, 1963 m a.s.l.), one female (16–302) and two males (16–303, 16–305) collected on 19 July 2016 by J. Reyes-Velasco and S. Boissinot north of Maji (6.2365°N, 35.5712°E, 1936 m a.s.l.), one female (16–326) and one male (16–313) collected on 20 July 2016 by J. Reyes-Velasco and S. Boissinot northeast of Machi (6.3780°N, 35.6659°E, 2063 m a.s.l.), two males (16–327, 16–329) collected on 20 July 2016 by J. Reyes-Velasco and S. Boissinot northeast of Mizan Teferi (7.0203°N, 35.7545°E, 2449 m a.s.l.), two females (SB333, SB334) collected on 9 June 2018 by S. Goutte and J. Reyes-Velasco northwest of Gore (8.2014°N, 35.3772°E, 1666 m a.s.l.), one male (SB388) collected on 11 June 2018 by S. Goutte and J. Reyes-Velasco south of Gech’a (7.5544°N, 35.4148°E, 1936 m a.s.l.), one female (SB405) collected on 13 June 2018 by S. Goutte and J. Reyes-Velasco south of Gech’a (7.5185°N, 35.4163°E, 1917 m a.s.l.), one female (SB462) collected on 20 June 2018 by S. Goutte and J. Reyes-Velasco northeast of Shebe (7.5423°N, 36.5732°E, 2240 m a.s.l.).

#### Diagnosis.

**Figure 23.**
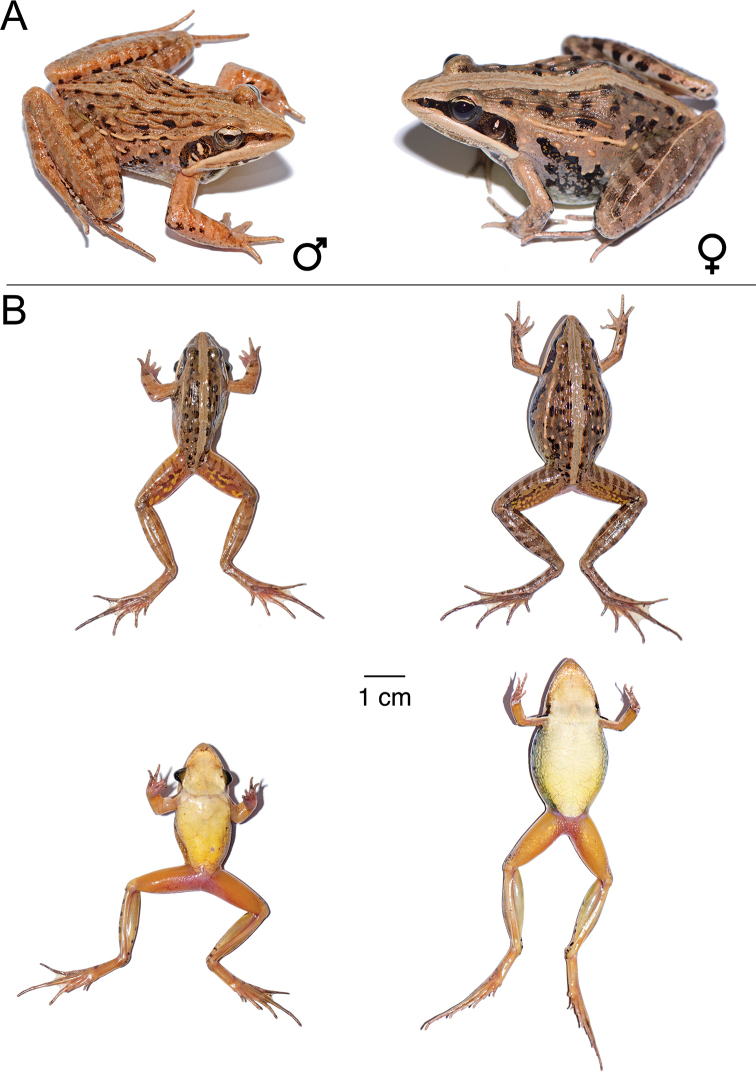
*Ptychadena neumanni***A** Live male (SB393; left) and female (SB388, right) **B** dorsal and ventral views of a male (SB333, left) and a female (SB388, right) after euthanasia and before fixation.

Medium-sized species (male (20) SVL 35.7 ± 2 mm, female (8) SVL 45.6 ± 1.4 mm) of the *neumanni* species group (Fig. 23) distinguished by the following combination of characters: (1) long hind limbs (male TL/SVL 0.58 ± 0.02, female TL/SVL 0.57 ± 0.01), (2) vertical cream bar on the tympanum, (3) vocal sacs uniformly dark grey.

#### Comparison.

Smaller than *P. cooperi* and *P. goweri* and larger than *P. nana* and *P. robeensis* sp. nov. Head wider than *P. erlangeri* and *P. levenorum* but narrower than *P. beka* sp. nov., Head and snout longer than *P. erlangeri* and *P. levenorum*. Wider inter-orbital distance than *P. doro* sp. nov., *P. beka* sp. nov., *P. erlangeri*, *P. levenorum*, and *P. amharensis*. Wider inter-nares distance and longer eye-nostril distance than *P. erlangeri* and *P. levenorum*. Tympanum larger than *P. doro* sp. nov., *P. erlangeri*, and *P. levenorum*. Larger hands than *P. erlangeri* and *P. levenorum*. Longer thighs and feet than *P. erlangeri*, *P. levenorum*, *P. amharensis*, and *P. beka* sp. nov.

#### Description of the lectotype.

**Figure 24.**
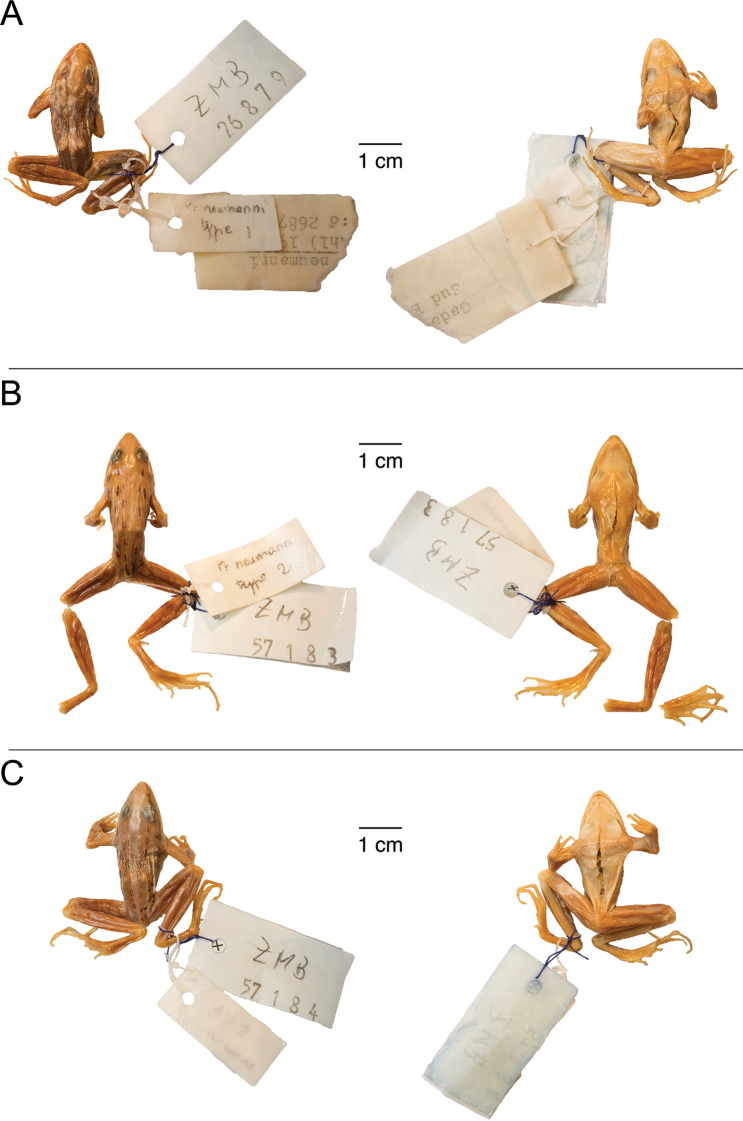
Type specimens of *Ptychadena neumanni*. Dorsal and ventral views of three male type specimens **A** ZMB26879–1, lectotype **B**ZMB–57183 = ZMB26879–2, paralectotype **C**ZMB–57184 = ZMB26879–3, paralectotype.

The lectotype is very desiccated (Fig. 24A) and the description of some characters is hindered by the state of conservation of the specimen. Medium sized (SVL 32.2 mm), slender adult male. Snout pointed, projecting beyond the lower jaw. Interorbital distance 0.83 × eye diameter. Head longer than wide (HW/HL 0.80). Nostril half-way between the tip of the snout and the eye. Internarial distance 1.1 × interorbital distance. Tympanum 0.64 × eye diameter. Finger tips not expanded but rounded, with moderate subarticular tubercles. Finger formula: I<II<IV<III. Hand free of webbing. Hindlimbs elongated, with tibia length 0.57 × snout-vent length. Foot longer than thigh (FL/THL 1.1) and as long as tibia. Toe tips rounded. Subarticular tubercles small and round. Inner metatarsal tubercle present, external absent. Toe formula: I<II<III<V<IV. Foot webbing formula: Ie(1), IIi/e(1–2), IIIi/e(2–2), IVi/e(2–2), Vi(2). Two light, continuous lateral ridges, six dorsal ridges difficult to see due to preservation of the specimen. No vertebral nor sacral ridges. Small warts on the body. No sacral, femoral, or crural folds.

#### Coloration of the lectotype in preservative.

Coloration has faded away with time and some patterns are now hardly distinguishable. Dorsal background color is brown, with irregular elongated dark brown blotches distributed along the dorsal ridges and on the antero-dorsal part of the flanks. A wide light vertebral line from snout to vent is present. Dark brown canthal stripe from the tip of the snout to the back of the jaw. Vocal sacs dark grey. Upper lip, throat, and posterior part of flanks cream. Ventrum, ventral side of the thighs and tibias uniformly cream in type 1. Barely distinguishable irregular dark brown bars on the tibias and on the thighs.

#### Variations.

In life, background color varies from light to dark brown. Dorsal ridges vary in number and definition. Lateral ridges may be sand or dark orange. All specimens examined had small dark brown or black blotches distributed symmetrically on the dorsal ridges. Flanks more or less heavily colored with black or dark brown blotches. Vertebral stripe absent in some individuals. When present, the vertebral stripe may be thin or wide, sand-color or brown-orange.

Iris bicolored, the upper third silver and lower two thirds dark brown sometimes marbled with light yellow or copper on the bottom half. Upper jaw cream, lower jaws featuring irregular light grey or brown markings but no barring. All specimens examined featured a dark brown canthal stripe from the tip of the snout to the back of the upper jaw and a with a cream vertical stripe. A thin cream longitudinal stripe on the tibia present in some individuals. Tibias, thighs, and feet posteriorly barred with more or less defined brown or light brown markings. Some individuals have dark irregular markings on the posterior side of the arms and anteroventral sides of the thighs and tibias. Thighs posteriorly marbled with dark brown and yellowish brown. Ventrum and throat uniformly cream to yellow. Vocal sacs grey to dark grey. Small warts over the back and flanks in adult males.

#### Habitat, distribution, and natural history.

*Ptychadena neumanni* is found on both sides of the Great Rift Valley (GRV) and limited to the south and southwestern highlands of Ethiopia (5.80–8.20°N, 35.36–38.64°E). This species is found at elevations ranging from 1409 m to 2449 m a.s.l. The southernmost individuals were found north of Hagere Mariam (east of the GRV) and Maji (west of the GRV). In the west, populations are found west of Gore, Gech’a, Bonga and Jimma. *Ptychadena neumanni* has also been found between Sodo and Bonga. East of the GRV it has been found in the vicinity of Wondo Genet. In the southwest, *P. neumanni* is found in syntopy with multiple *Ptychadena* species: *P. doro* sp. nov., *P. delphina* sp. nov., *P. beka* sp. nov., and the lowland species *P. anchietae*. In the southeast, it is found in sympatry with *P. goweri*. Males are found calling at night in shallow puddles on or beside the road, or in flooded grassy meadows.

#### Advertisement call.

*Ptychadena neumanni* produces two types of call, hereafter referred to as call type A and call type B. Based on our video recordings and observations in the field, we believe that call type A corresponds to the advertisement call, while call type B may be a territorial call. Notably, while calling in chorus, males were producing call type A, whereas call type B seem to be employed in two-individuals vocal battles. However, our data are insufficient to categorize with confidence these two calls with regard to their respective function and we describe both call types below. The call type and call rate produced is highly dependent on the social context.

*Ptychadena neumanni* call type A (3 males, 78 calls) is 307 ± 149 ms long and contains 5.8 ± 2.4 pulsed notes. Notes are 32 ± 14 ms in duration and are produced at regular intervals (19.1 ± 3.5 notes s^-1^) within each call. Amplitude modulation is very low within the call. Call type A’s dominant frequency is 2406 ± 339 Hz, with no frequency modulation within notes or calls.

*Ptychadena neumanni* call type B (1 male, 13 calls) is composed of one initial long (437 ± 54 ms) pulsed note (note B1), followed by 3 ± 0.6 distinct pulses (note B2). The peak frequency of note B1 is 2207 ± 129 Hz while note B2 has a dominant frequency of 2337 ± 191 Hz.

Call type A of *P. neumanni* can be distinguished from those of all other species of the P. neumanni complex, except for *P. cooperi* and *P. amharensis*, by its composition of multiple pulsed notes with indistinguishable pulses. It differs from the call of *P. cooperi* and *P. amharensis* by the absence of frequency modulation, shorter notes, and shorter inter-note intervals.

Call type B of *P. neumanni* is unique within the *P. neumanni* complex in its composition of two different types of notes and can easily be distinguished from those of all other species of the group.

#### Remarks.

Ahl (1924) described *Rana neumanni* based on 35 syntypes collected in Didda (one specimen), Somaliland (23 specimens), Gadat (Gofa) (three specimens), and Uba (eight specimens). Perret (1980) examined the type series, split the collection, and assigned the specimens to three distinct species. Perret designated the three specimens from Gadat (ZMB–26879 type 1, ZMB–26879 type 2 and ZMB–26879 type 3) as syntypes of *Ptychadena neumanni* (sensu stricto) as they were the only specimens with individual tags. Additionally, those specimens were the only ones of the original type series to have a collection date: 2 February 1901. Gadat (Gofa), south Ethiopia thus became Terra typica restricta of *Ptychadena neumanni*. Perret (1980) then revised the description of the species and gave measurement values for the three syntypes. When we examined the type series in the collection of the Museum of Berlin, we realized that the syntypes 2 and 3 had been attributed new collection numbers: ZMB–57183 (ZMB–26879 type 2) and ZMB–57184 (ZMB–26879 type 3) and the jar containing ZMB–26879 type 1 bears the label “*lectotypus*”. To our knowledge, no designation of ZMB–26879 type 1 as lectotype for *P. neumanni* has been published. Given that the original description of the species by Ahl (1924) included 35 syntypes and that the restriction by Perret (1980) is not valid according to the Code as it does not designate an individual specimen, we hereby designate ZMB–26879 (type 1) as a lectotype according to Article 74 of the International Zoological Code of Nomenclature (ICZN 1999). The specimens ZMB–57183 (ZMB–26879 type 2) and ZMB–57184 (ZMB–26879 type 3) thus become paralectotypes.

## Discussion

The recent efforts made towards a biological inventory of Ethiopia demonstrate how species diversity in the Ethiopian highlands is likely to be largely underestimated (e.g., Goutte et al. 2019; Koppetsch 2020; Kostin et al. 2020; Soultan et al. 2020) and taxonomic revision of multiple groups is urgently needed to truly appreciate the ecological importance of this region. Describing the diversity of groups such as the genus *Ptychadena* is, however, challenged by the morphological resemblance of the species. In this study, we combined morphometrics, bioacoustics, and genetics to untangle the diversity of *Ptychadena* in the highlands of Ethiopia. Our integrative analysis distinguished a total of 12 species in the *Ptychadena neumanni* complex, four of which are new to science.

As in any taxonomic work, an important step in resolving the nomenclatural issues of the *P. neumanni* complex was the comparison of the newer material with the type series of the previously described species of the group. Although in the case of Ethiopian *Ptychadena* we were able to extract DNA from formalin-preserved type specimens and include those sequences in phylogenetic analyses (Reyes-Velasco et al. in review), this is not always possible. A careful morphological examination of all the material available thus often remains the only way to compare century-old specimens to more recently collected material and should not be overlooked.

As in other studies on African grass frogs of the genus *Ptychadena*, advertisement calls provided better discriminant characters than morphometry (e.g., Bwong et al. 2009; Dehling and Sinsch 2013). In the field, syntopic species may not be readily distinguishable morphologically (e.g., *P. delphina* sp. nov. and *P. doro* sp. nov.), however, their advertisement calls easily allow their identification. We therefore provide an acoustic key to the *Ptychadena* of Ethiopian highlands in the hope that it will be used by ecologists and conservationists as a cost-effective, non-invasive identification tool.

Information on species’ distribution patterns is essential in designing relevant conservation measures. The split of several species (e.g., *P. nana*, *P. neumanni*, and *P. erlangeri*) often resulted in splitting their distribution ranges. Our results also revealed important differences in sizes of the distribution range among species of the *P. neumanni* complex. For example, *P. robeensis* sp. nov. occupies a very reduced area (20 × 20 km, Fig. 5B), while *P. erlangeri* occurs on both sides of the Great Rift Valley (Fig. 5A; Freilich et al. 2016). These distribution patterns thus call for conservation measures spread across the Ethiopian highlands rather than a focus on only a few hotspots.

## Supplementary Material

XML Treatment for Ptychadena amharensis

XML Treatment for Ptychadena erlangeri

XML Treatment for Ptychadena levenorum

XML Treatment for Ptychadena nana

XML Treatment for Ptychadena robeensis

XML Treatment for Ptychadena beka

XML Treatment for Ptychadena delphina

XML Treatment for Ptychadena doro

XML Treatment for Ptychadena goweri

XML Treatment for Ptychadena neumanni

## Supplementary materials

Supplementary material 1 Appendix S1 Data type identification key Explanation note Key to the species of Ptychadena in the Ethiopian highlands. https://binary.pensoft.net/file/508441This dataset is made available under the Open Database License (http://opendatacommons.org/licenses/odbl/1.0/). The Open Database License (ODbL) is a license agreement intended to allow users to freely share, modify, and use this Dataset while maintaining this same freedom for others, provided that the original source and author(s) are credited.Sandra Goutte, Jacobo Reyes-Velasco, Xenia Freilich, Abeje Kassie, Stephane Boissinot

Supplementary material 2 Appendix S2 Data type call identification key Explanation note Key to the calls of the Ptychadena neumanni species complex. https://binary.pensoft.net/file/508442This dataset is made available under the Open Database License (http://opendatacommons.org/licenses/odbl/1.0/). The Open Database License (ODbL) is a license agreement intended to allow users to freely share, modify, and use this Dataset while maintaining this same freedom for others, provided that the original source and author(s) are credited.Sandra Goutte, Jacobo Reyes-Velasco, Xenia Freilich, Abeje Kassie, Stephane Boissinot

Supplementary material 3 Tables 1–6 Data type excel file Explanation note **Table S1.** Morphometric measurements and localities of all examined individuals. **Table S2.** GenBank accession numbers. **Table S3.** Morphometric measurements ratios (average ± standard deviation) of the Ptychadena neumanni complex. **Table S4.** Results of discriminant analyses based on linear morphometric measurements of recently sampled specimens of the P. neumanni complex. **Table S5.** Summary table presenting the results of the Tukey HSD pairwise tests based on linear morphometrics of the Ptychadena neumanni complex. **Table S6.** Taxonomic history of the Ptychadena neumanni species complex. https://binary.pensoft.net/file/508443This dataset is made available under the Open Database License (http://opendatacommons.org/licenses/odbl/1.0/). The Open Database License (ODbL) is a license agreement intended to allow users to freely share, modify, and use this Dataset while maintaining this same freedom for others, provided that the original source and author(s) are credited.Sandra Goutte, Jacobo Reyes-Velasco, Xenia Freilich, Abeje Kassie, Stephane Boissinot
