# Supplementary material for: Taxonomic revision of grass frogs (Ptychadenidae, Ptychadena) endemic to the Ethiopian highlands
Source: Zookeys. 2021 Feb 11;1016:77–141. doi: 10.3897/zookeys.1016.59699 (PMC7892535; doi:10.3897/zookeys.1016.59699)
Supplement: Supplementary material 1 — Appendix S1 [file zookeys-1016-077-s001.docx]

# Appendix 1. Key to the species of *Ptychadena* in the Ethiopian highlands

**1a.** large body size (male SVL > 45 mm, female SVL > 55 mm), dorsal folds formed by rows of short ridges, foot webbing extending beyond the second phalange of the fourth and fifth toe, dark grey vocal sacs ………..…………………………………………………...…...…………………..*P. cooperi*

**1b.** very small (male SVL < 32 mm, female SVL < 34.5 mm), short hind limbs (male THL/SVL 0.37-0.4, female THL/SVL 0.33–0.47), foot webbing extending at most to the second phalange of the fourth toe, vocal sacs at least partially pigmented with grey …….…………...……**2**

**1c.** small to large body size (male SVL 31–47 mm, female SVL 33–56 mm), foot webbing extending at most to the second phalange of the fourth toe ………….………………………….…………**3**

**2a.** outer metatarsal tubercle present, dorsal ridges uninterrupted except in the sacral region; back of the thigh with a pattern of dark streaks on pale background..................*P. wadei*

**2b.** outer metatarsal tubercle absent, back of the thighs molted or marbled dark and light brown or cream..........................................................................................................................................................**4**

**3a.** no stripe or blotch on the tympanum, vocal sacs yellow or cream, adult males with thickened forearms……………………………………………………………………………..….......…...*P. erlangeri*

**3b.** long hind limbs (male TL/SVL 0.52–0.77, female TL/SVL 0.55–0.85; male THL/SVL 0.42–0.64, female THL/SVL 0.43–0.80) …………………….………………………………....................….………..…..**5**

**3c.** short hind limbs (male TL/SVL 0.37–0.56, female TL/SVL 0.44–0.61; male THL/SVL 0.33–0.5, female THL/SVL 0.40–0.52) …………………………………..…….……...……………..........….……**6**

**4a.** no stripe or blotch on the tympanum, small warts almost always present in adult males during the breeding season ..............................................……………………….....……..……………..…*P. nana*

**4b**. light blotch on the tympanum, no warts ………..………..…………….............….…....……*P. robeensis*

**5a.** Large body size (male SVL 36.6–47 mm, female SVL 46.4–55.4 mm), long hind limbs (male TL/SVL 0.57–0.77, female TL/SVL 0.57–0.62), well developed metatarsal tubercles and relatively large toe discs, light vocal sacs ...…………………………………………...…..…......*P. goweri*

**5b.** vertebral stripe absent and only few faint dark markings on the back, dark grey vocal sacs….……..……………..…………………………………………..……………………….………….………....*P. harenna*

**5c.** tympanum small (male TD/ED 0.44–0.81, females TD/ED 0.49–0.8), eyes close from one-another (males IND/IOD 0.91–1.43, females IND/IOD 0.95–1.48), vocal sacs dark grey or cream and grey …………………………...…………………………………………….…………....…..................*P. doro*

**5d.** tympanum larger (male TD/ED 0.56–0.88, female TD/ED 0.60–1.0), eyes further apart (male IND/IOD 0.84–1.16, female IND/IOD 0.85–1.31), vocal sacs dark grey..........*.P. neumanni*

**6a.** small body size (male SVL 31–38 mm, female SVL 34.5–40.5 mm), snout short (male SL/SVL 0.09–0.15, female SL/SVL 0.12–0.14), vocal sacs most often bicolor, cream and grey …….….…………………………………………………………………………………………….………..........*P. levenorum*

**6b**. Body size medium to large (male SVL 33.8–44.2 mm, female SVL 38.4–49.2 mm), snout longer (male SN/SVL 0.14–0.17, female SL/SVL 0.13–0.17) ………………...…………..........................**7**

**7a**. eyes close to one another (male IOD/ED 0.42–0.61, female IOD/ED 0.43–0.69), vocal sacs light grey or cream, males covered with small warts during the breeding season………….........… …………………………………………………………………………………………………………......……..*P. amharensis*

**7b.** eyes further apart (male IOD/ED 0.51–0.86, female IOD/ED 0.52–1.0), vocal sacs at least partially dark grey …………………………………….………………………….…..…………...……………….............**8**

**8a.** forearm moderate in size (FLL/SVL 0.16–0.20) and not thickened in adult males, vocal sacs bicolor cream and grey, warts almost always absent in males …….……...…..…………..*P. beka*

**8b**. forearms relatively long (FLL/SVL 0.19–0.22), vocal sacs dark grey or bicolor, cream and dark grey …………………………………………..………………………..……….……………………..........*P. delphina*
