# Supplementary material for: Taxonomic revision of grass frogs (Ptychadenidae, Ptychadena) endemic to the Ethiopian highlands
Source: Zookeys. 2021 Feb 11;1016:77–141. doi: 10.3897/zookeys.1016.59699 (PMC7892535; doi:10.3897/zookeys.1016.59699)
Supplement: Supplementary material 2 — Appendix S2 [file zookeys-1016-077-s002.docx]

# Appendix 2. Key to the calls of the *Ptychadena neumanni* species complex

**1a.** call composed of one or several pulsed notes, with indistinct or partly fused pulses........**2**

**1b.** call composed of one note with distinct pulses...................................................................................**3**

**1c.** call composed of one initial, indistinctively pulsed note followed by 2–4 distinct pulses... ..............................................................................................................................................*P. neumanni* call type B

**2a.** call composed of a single note of 370–450 ms duration, with increasing dominant frequency from 1752 ± 87 Hz to 1999 ± 93 Hz................................................................................*P. doro*

**2b.** frequency-modulated notes with and increased frequency at the end of the note.............**4**

**2c.** 3–9 notes per call of 32 ± 14 ms duration and 2406 ± 339 Hz, without any frequency modulation ....................................................................................................................*.P. neumanni* call type A

**3a.** pulses at regular, relatively long (52–72 ms) intervals....................................................................**5**

**3b.** pulses separated by short inter-pulses intervals (7–20 ms) and forming pulses groups within the notes.........................................................................................................................................................**6**

**4a.** call dominant frequency 1653 ± 64 Hz...................................................................................*P. cooperi*

**4b.** call dominant frequency 2224 ± 158 Hz........................................................................*P. amharensis*

**5a.** call dominant frequency 2327 ± 147 Hz..............................................................................*P. delphina*

**5b.** call dominant frequency 2876 ± 74 Hz...............................................................................*P. robeensis*

**6a.** calls composed of 8–17 groups of 1–6 pulses ......................................................................................**7**

**6b.** calls composed of 3–9 groups of 3–33 pulses......................................................................................**8**

**6c.** short calls (< 300 ms) composed of 1–3 groups of 7.2 ± 4.7 pulses, call dominant frequency 2801 ± 52 Hz.............................................................................................................................*P. nana*

**7a.** long calls (> 550 ms) composed of 12–17 groups of 2–5 pulses (rarely one isolated pulse), call dominant frequency 2318 ± 86 Hz ...........................................................................*P. goweri*

**7b.** shorter call (< 550 ms) composed of 8–13 groups of 2–6 pulses, call dominant frequency 2482 ± 142 Hz...........................................................................................................................*P. beka*

**8a**. relatively long calls (> 370 ms) composed of 5–9 groups of 10–33 pulses, call dominant frequency 2223 ± 90 Hz .................................................................................................................*P. levenorum*

**8b.** shorter calls (< 325 ms) composed of 3–6 groups of 3–21 pulses, call dominant frequency 2343 ± 454 Hz .................................................................................................................*P. erlangeri*
